# A new genus (*Durabilispora*) and two new species (*D. carpatica*, *Dominikia tatrensis*) in *Glomerales (Glomeromycota)*

**DOI:** 10.3897/mycokeys.134.187344

**Published:** 2026-06-24

**Authors:** Janusz Błaszkowski, Paweł Milczarski, Ryszard Malinowski, Piotr Niezgoda, Bruno Tomio Goto, Anna Ronikier, Małgorzata Stanek, Paulina Janik, Szymon Zubek

**Affiliations:** 1 Department of Environmental Management, West Pomeranian University of Technology in Szczecin, Słowackiego 17, PL–71434 Szczecin, Poland Institute of Botany, Faculty of Biology, Jagiellonian University Kraków Poland; 2 Department of Genetic, Plant Breeding & Biotechnology, West Pomeranian University of Technology in Szczecin, Słowackiego 17, PL–71434 Szczecin, Poland Departamento de Botânica e Zoologia, Universidade Federal do Rio Grande do Norte, Campus Universitário Natal Brazil; 3 Departamento de Botânica e Zoologia, Universidade Federal do Rio Grande do Norte, Campus Universitário, 59078–900, Natal, RN, Brazil Department of Environmental Management, West Pomeranian University of Technology in Szczecin Szczecin Poland; 4 W. Szafer Institute of Botany, Polish Academy of Sciences, Lubicz 46, 31-512 Kraków, Poland Department of Genetic, Plant Breeding & Biotechnology, West Pomeranian University of Technology in Szczecin Szczecin Poland; 5 Institute of Botany, Faculty of Biology, Jagiellonian University, 30–387, Kraków, Poland W. Szafer Institute of Botany, Polish Academy of Sciences Kraków Poland

**Keywords:** 45S nuc rDNA, arbuscular mycorrhizal fungi, morphology, phylogenetic taxonomy, *rpb*1

## Abstract

Morphological studies, as well as comparisons and phylogenetic analyses of sequences of the 45S (= 18S-ITS-28S) nuc rDNA region and the largest subunit of the RNA polymerase II (*rpb*1) gene of two specimens, preliminarily named Isolate 527 and Isolate 530, showed that they are undescribed species in a newly proposed genus of the family *Rhizoglomeraceae* and in the genus *Dominikia* in *Dominikiaceae* (order *Glomerales*, phylum *Glomeromycota*), respectively. Consequently, Isolate 527 was described as *Durabilispora carpatica***sp. nov**. in *Durabilispora***gen. nov**., and Isolate 530 as *Dominikia tatrensis***sp. nov**. *Durabilispora* was previously identified as an undescribed genus informally named “gen21” by other researchers. Both new species produced glomoid glomerospores in glomerocarps, which were extracted from trap cultures inoculated with mixtures of rhizosphere soils and root fragments of meadow plants from the Tatra Mountains, southern Poland. The morphological identity of *Du. carpatica* to the previously found Isolate 299, which was not provided with genetic data, indicated that *Du. carpatica* also inhabited dunes of the Baltic Sea in northern Poland.

## Introduction

*Glomerales* is one of the nine orders of the phylum *Glomeromycota*, comprising arbuscular mycorrhizal fungi (AMF), that produce glomoid spores sensuMorton and Redecker (2001). This order consists of nine families and 26 genera and is the species-richest taxon at this rank in *Glomeromycota* (da Silva et al. 2024; Oehl et al. 2026). Among the genera of this order are *Rhizoglomus* and *Dominikia*, whose members were considered in the present study.

*Rhizoglomus*, with the type species *R. intraradices*, was established by Sieverding et al. (2014) and includes species that Schüßler and Walker (2010) and Walker et al. (2021) classified in the genus *Rhizophagus*, with the type species *R. populinus*. The arguments of Sieverding et al. (2014) were accepted, and hereafter, the name *Rhizoglomus* is used instead of *Rhizophagus*. da Silva et al. (2024) placed *Rhizoglomus* in the family *Sclerocystaceae*, the most taxonomically diverse family in *Glomeromycota* (Wijayawardene et al. 2025), and Oehl et al. (2026) accommodated *Rhizoglomus* in a newly described family, *Rhizoglomeraceae*.

The morphological and ecological features distinguishing most, but not all, *Rhizoglomus* species are the relatively frequent formation of intraradical spores, with the main structural laminate spore wall layer composed of easily separable sublayers (laminae) in crushed spores (Schüßler and Walker 2010; Błaszkowski 2012; Sieverding et al. 2014). Exceptions are, e.g., *R. invermaium* and *R. melanum*, in which the laminate spore wall layer in crushed spores usually remains intact (Błaszkowski 2012; Sudová et al. 2015). da Silva et al. (2024) and Oehl et al. (2026) stated that *Rhizoglomus* species are also distinguished by the formation of spore subtending hyphae, the pore of which is relatively wide and usually open. Currently, *Rhizoglomus* includes 17 species with known phylogenies.

The genus *Dominikia*, with the type species *D. minuta*, was established in the family *Glomeraceae sensu* Piroz. et Dalpé of *Glomeromycota* based on phylogenetic analyses of 45S nuc rDNA (= 18S-ITS-28S) sequences (Błaszkowski et al. 2015a). The analyses included six species originally described in the genus *Glomus*, the newly described *Dominikia disticha*, and an isolate called *Dominikia* 211, which was recently newly described as *D. paraminuta* (Błaszkowski et al. 2025). Until recently, *Dominikia sensu*Błaszkowski et al. (2015a) included 17 species. Corazon-Guivin et al. (2019a) transferred *D. emiratia*, described by Al-Yahya’ei et al. (2017), and *D. litorea*, described by Błaszkowski et al. (2018), to new genera, *Orientoglomus*, with *O. emiratium* comb. nov., and *Microdominikia*, with *M. litorea* comb. nov., respectively. da Silva et al. (2024) placed *Dominikia* in a newly described family, *Dominikiaceae*, and transferred *D. compressa* to *Macrodominikia* gen. nov. as *M. compressa* comb. nov. in *Dominikiaceae*. Błaszkowski et al. (2025) established a new genus in *Dominikiaceae*, *Delicatispora*, with *De. indica* comb. nov., which was originally described in *Glomus* under the name *G. indicum* (Błaszkowski et al. 2010), then transferred to *Dominikia*, and renamed *D. indica* comb. nov. Currently, *Dominikia* consists of 14 species that produce spores in loose to compact clusters without peridium, with the exception of *D. aurea*, *D. difficilevidera*, and *D. glomerocarpica*. *Dominikia difficilevidera* produces spores singly in the soil, while spores of *D. aurea* and *D. glomerocarpica* are formed in compact hypogeous and epigeous glomerocarps, respectively (Błaszkowski 2012; Błaszkowski et al. 2015b, 2021a). The spores of most *Dominikia* species are colorless or lightly colored and very small (12–86 µm diam when globose).

The state of knowledge of the species richness of *Glomeromycota* is poor. Estimates based on environmental sequences showed that the approximately 370 described species constitute only 0.04–4.9–36.0% of all species of this phylum existing in the world (Hyde et al. 2023; Lutz et al. 2025). Importantly, they also showed that among these undescribed AMF, there are numerous taxa belonging and closely related to *Dominikia*.

Stefani et al. (2025) showed that all rDNA loci used in studies dealing with the identification and classification of members of *Glomeromycota* are polymorphic and can lead to erroneous interpretations of closely related species. This risk can be greatly reduced when such inferences are additionally based on protein-coding gene sequences, which are characterized by much lower variability (Stockinger et al. 2014), and concatenated sequences of both locus groups, provided there is no conflict between the phylogenetic results. Of the three protein-coding gene sequences used in phylogenetic studies of *Glomeromycota*, those of the largest subunit of RNA polymerase II (*rpb*1) gene have been obtained for by far the largest number of species (Stefani et al. 2025). Most of the sequences, amplified by primers designed by Stockinger et al. (2014), consist of five exons and four introns. Stockinger et al. (2014) found that (i) among these components, the number of diagnostic nucleotides was the highest in intron 1, exon 3, and the analyzed part of exon 5, and (ii) the *rpb*1 gene showed an excellent barcode gap between species. Stefani et al. (2025) reconstructed *rpb*1 phylogenies of *Glomeromycota* members based on sequences of only exon 4 plus exon 5 and found that most of the analyzed species differed when their pairwise sequence distances were ≥ 1.1%, although exons 4 plus 5 had no global barcode gap.

The Tatra Mountains in southern Poland and northern Slovakia are part of the Carpathians, one of the most important mountain massifs in Europe, with occurrences of many rare plant species (Mirek 1996; Piękoś-Mirkowa et al. 1996). Extensive mycological studies conducted by Dominik and colleagues (Dominik 1961) revealed the common occurrence of arbuscular mycorrhiza in the plant communities of the Polish part of the Tatras. However, these studies did not take into account the species of AMF that formed this mycorrhiza. The reason was that the method of collecting these fungi by wet sieving and decanting of rhizosphere soils (Gerdemann and Nicolson 1963), which is widely used today, was not known at that time. Studies by Błaszkowski (2012) and Zubek et al. (2005, 2008, 2009a, 2009b) also showed the commonness of arbuscular mycorrhiza in Tatra plants and revealed the presence of 26 species of AMF in this region.

Thirty-two trap cultures inoculated with mixtures of rhizosphere soils and root fragments of plants growing in the Tatra Mountains were examined. Among the AMF specimens extracted from these cultures, there were two, preliminarily named Isolate 527 and Isolate 530, whose distinctive morphology suggested that they were undescribed members of *Glomeromycota*. To confirm this suggestion, morphological analyses of these specimens were performed, and their phylogenies were reconstructed based on sequences of the 45S nuc rDNA region and the *rpb*1 gene. This study also aimed to reveal genetic differences determined by comparisons of the molecular content of exons and introns present in the *rpb*1 sequences of the species investigated in this study.

## Materials and methods

### Origin of study material

Both isolates were characterized here based on specimens extracted from two trap pot cultures because numerous attempts to grow Isolate 527 and Isolate 530 in single-species cultures failed. The trap cultures contained approximately 500 mL of autoclaved quartz sand inoculated with 50 g of fresh field mixtures of rhizosphere soil and root fragments of meadow plants from the Tatra Mountains, southern Poland. The surface of these cultures was sown with approximately 20 seeds of *Plantago lanceolata* L. as the host plant. The cultures were placed in closed Sun bags (Sigma-Aldrich) to avoid contamination, grown for 6 months in a plant growth chamber at 22 °C, 345 µmol PAR photons m^-2^ s^-1^, 12/12 h light regime, and watered with 50 mL of distilled water once a week. The inocula with Isolate 527 and Isolate 530 were sampled by Anna Ronikier and Paulina Janik on July 18 and May 11, 2023, respectively. The inoculum containing Isolate 527 came from the Tatra 1/4op site located in the lower part of the Dolina Miętusia Valley, at an altitude of 938 m asl (49°5'49.25"N, 19°52'17.33"E) in the lower montane climatic-vegetation zone. The site was a windfall open area located near a forest and a grazing meadow (for details on the plant composition of this site and the Isolate 530 site, see Suppl. material 6). The inoculum with Isolate 530 was collected at the Tatra 2/1op site located in the northwestern part of the Polana Upłaz meadow, at the edge of the spruce forest, 1298 m asl (49°15'3.6"N, 19°52'46.92"E), in the upper montane climatic-vegetation zone. The chemical properties of the field soil samples with Isolate 527 and Isolate 530 (here reported in pairs separated by a slash) were as follows: pH, 5.17/6.85; total carbon, 7.186/14.764%; N, 0.4773/1.1725%; total P, 135.52/168.49 mg/kg; total Ca, 3362.1/15502 mg/kg. The climate type in the Tatras is cool temperate moist (Sayre et al. 2020). Spores and mycorrhizal structures from trap cultures were extracted and stained as described previously (Błaszkowski et al. 2006, 2009).

Additional study material included specimens named Isolate 299, whose morphology was identical to that of Isolate 527. Isolate 299 was found in a field sample of the rhizosphere soil of *Rosa rugosa* Thunb., which inhabited the Baltic Sea dunes near Jastarnia (54°41'58"N, 18°40'36"E), northern Poland. The soil sample was collected by J. Błaszkowski on 5 September 2015. This fungus has not been described because attempts to obtain its genetic data were unsuccessful.

### Microscopy and nomenclature

Morphological features of glomerocarps and glomerospores, as well as phenotypic and histochemical characters of spore wall layers of Isolate 527 and Isolate 530 were characterized based on at least 20 glomerocarps and 50–100 spores mounted in water, lactic acid, polyvinyl alcohol/lactic acid/glycerol (PVLG, Omar et al. 1979), and a mixture of PVLG and Melzer’s reagent (1:1, v/v). Spores for study and photography were prepared as described in Błaszkowski (2012) and Błaszkowski et al. (2012). The types of spore wall layers were defined by Błaszkowski (2012) and Walker (1983). Color names were from Kornerup and Wanscher (1983). Nomenclature of fungi and the authors of fungal names are from the Index Fungorum website https://indexfungorum.org. The terms “glomerospores” and “glomerocarps” were used for spores and sporocarps produced by AMF, as proposed by Goto and Maia (2006) and Jobim et al. (2019).

The holotypes of the new species were deposited at ZT Myc (ETH Zurich, Switzerland), and their isotypes were deposited in the Laboratory of Plant Protection, Department of Environmental Management (LPPDEM), West Pomeranian University of Technology in Szczecin, Poland. In all specimens, spores were permanently mounted in PVLG and a mixture of PVLG and Melzer’s reagent (1:1, v/v) on slides.

### DNA extraction, PCR, cloning, and DNA sequencing

Genomic DNA of Isolate 527 and Isolate 530 was separately extracted from eight fragments of glomerocarps, each with ca. 5–20 spores. The method of processing the spores prior to PCR and the conditions and primers used for PCR to obtain 45S sequences of the isolates were as described by Krüger et al. (2009) and Błaszkowski et al. (2021a). To obtain *rpb*1 sequences of both isolates, PCRs were performed under conditions recommended by and with the primers RPB1-HS_A1a and RPB1-DR1730rr designed by Stockinger et al. (2014). Cloning and sequencing of PCR products to obtain both types of sequences were performed following the protocols described by Błaszkowski et al. (2021a). The sequences were deposited in GenBank (PX641487–PX641499, PX570049–PX570054).

### Phylogenetic analyses

Preliminary comparisons of 45S sequences of Isolate 527 and Isolate 530 with sequences of this region or its part, available in GenBank, showed that they represent different taxa of *Glomerales*. To determine the position and taxonomic status of these fungi, an alignment containing 45S sequences or parts thereof was prepared using MAFFT v. 7 with the E-INS-i option (Katoh et al. 2019). The sequences characterized representative species of all genera of *Glomerales*, except for *Parvocarpum badium* and *Simiglomus hoi* (see “Discussion” for the reasons). The outgroup of this alignment comprised sequences of *Entrophospora claroidea* (*Entrophosporaceae*), as *Entrophosporales* was indicated to be a sister order to *Glomerales* (Montoliu-Nerin et al. 2021; Błaszkowski et al. 2022). The alignment, divided into five partitions (18S, ITS1, 5.8S, ITS2, 28S), was subjected to Bayesian inference (BI) and maximum likelihood (ML) phylogenetic analyses, performed according to the principles given below. These analyses suggested that Isolate 527 should represent a new genus located below a clade with sequences of *Rhizoglomus* species and above a two-genus clade with *Sclerocystis* and *Silvaspora* (Suppl. material 1). These analyses also showed that Isolate 530 nested in a clade sister to the clade with *Dominikia aurea* and that the closest relatives of the *Dominikia* clade were members of the genera *Complexispora*, *Glomus*, and *Sclerocarpum* (Suppl. material 1). To confirm these indications and suggestions, six further alignments were assembled. All reference sequences used in this study are listed in Suppl. material 8.

**Figure 1.**
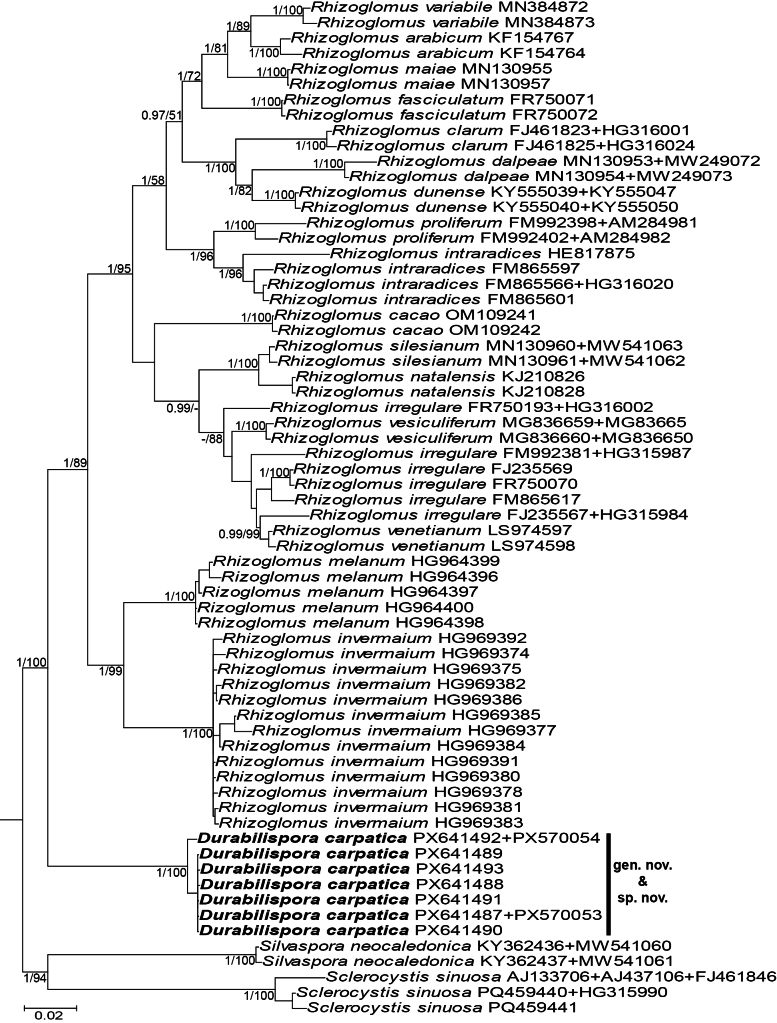
A 50% majority-rule consensus tree from the Bayesian analysis of 45S nuc rDNA sequences concatenated with *rpb*1 sequences of *Durabilispora carpatica* (= Isolate 527) and 17 *Rhizoglomus* species representing the ingroup, as well as *Sclerocystis sinuosa* and *Silvaspora neocaledonica*, serving as the outgroup. The new genus and species are in bold font. The Bayesian posterior probabilities ≥ 0.90 and ML bootstrap values ≥ 50% are shown near the branches, respectively. Bar indicates a 0.02 expected change per site per branch.

**Figure 2.**
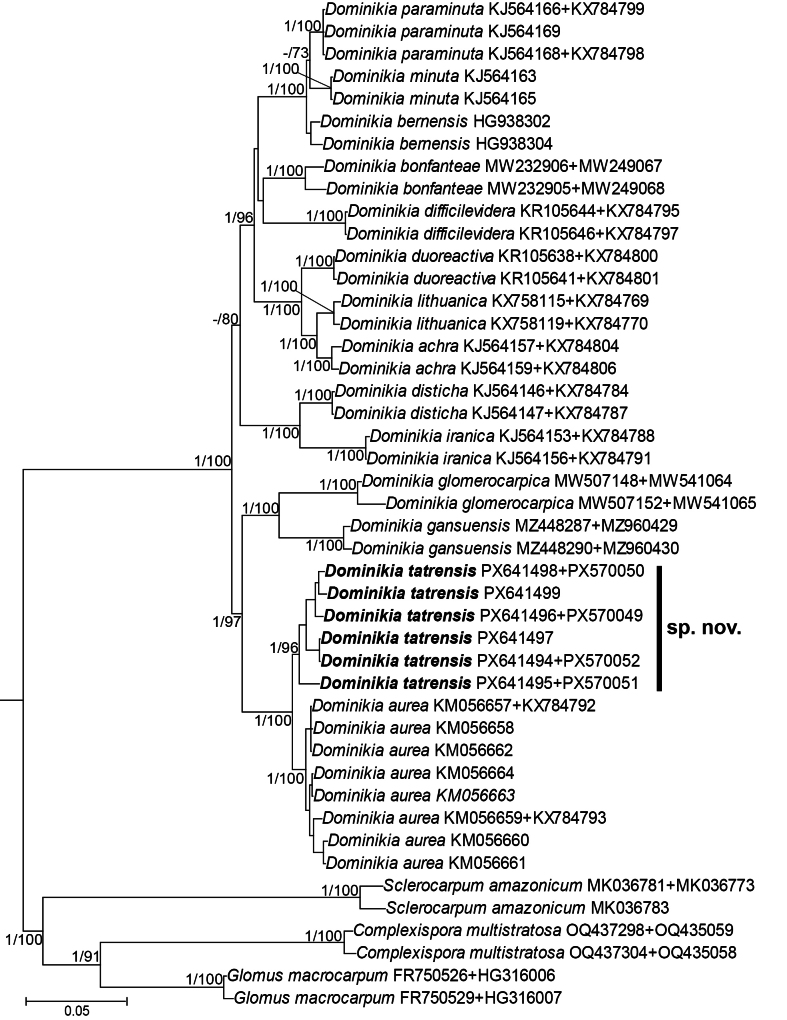
A 50% majority-rule consensus tree from the Bayesian analysis of 45S nuc rDNA sequences concatenated with *rpb*1 sequences of *Dominikia tatrensis* (= Isolate 530) representing the ingroup and 13 other *Dominikia* species, as well as *Complexispora multistratosa*, *Glomus macrocarpum*, and *Sclerocarpum amazonicum* serving as the outgroup. The new species is in bold font. The Bayesian posterior probabilities ≥ 0.90 and ML bootstrap values ≥ 50% are shown near the branches, respectively. Bar indicates a 0.05 expected change per site per branch.

In the 45SR alignment (R refers to the ingroup represented mainly by *Rhizoglomus* species), the ingroup contained 61 sequences of the 45S region or its part, which characterized Isolate 527 and 17 *Rhizoglomus* species (Suppl. material 2). The outgroup was sequences of *Sc. sinuosa* and *Si. neocaledonica* because they populated the closest generic clades in the 45S tree (Suppl. material 1). In the 45SD alignment (D refers to the *Dominikia* ingroup), the ingroup consisted of 39 45S sequences or their parts, which belonged to Isolate 530 and 13 *Dominikia* species (Suppl. material 3). The outgroup constituted sequences of *C. multistratosa*, *G. macrocarpum*, and *Scl. amazonicum*. The ingroup of the *rpb*1R alignment contained 28 sequences, of which two came from Isolate 527, while the others characterized all *Rhizoglomus* species sequenced from the *rpb*1 gene (Suppl. material 4). The outgroup was the *rpb*1 sequences of *Sc. sinuosa* and *Si. neocaledonica*. The ingroup of the *rpb*1D alignment comprised 37 sequences, including four that characterized Isolate 530, and the others came from *Dominikia* species (Suppl. material 5). The outgroup consisted of *rpb*1 sequences of *C. mediterranea*, *C. multistratosa*, *G. macrocarpum*, and *Scl. amazonicum*. The 45SR+*rpb*1R and 45SD+*rpb*1D alignments contained all sequences of the 45SR and 45SD alignments concatenated with sequences of the *rpb*1R and *rpb*1D alignments, respectively (Figs 1, 2).

To determine differences between sequences of exons and introns of the *rpb*1 gene of Isolate 527 and Isolate 530 and their closest relatives provided with *rpb*1 sequences and to compare these differences with those between 45S sequences of the same isolates and other *Glomeromycota* representatives, as exemplified by five *Glomus* species (Suppl. material 7), 17 alignments were created. Eleven of them were extracted from the *rpb*1R and *rpb*1D alignments mentioned above, and six were newly created with *rpb*1 and 45S sequences of the *Glomus* species. In the comparisons of exons and introns vs. 45S of Isolate 527 and Isolate 530, the *rpb*1R and *rpb*1D and 45SR and 45SD alignments were used, respectively.

The percentage sequence divergences of Isolate 527 and Isolate 530 from sequences of their closest relatives and other species analyzed were calculated in BioEdit (Hall 1999). All comparisons were performed on sequences of the same length.

The phylogenetic positions of Isolate 527 and Isolate 530 among the species present in the alignments mentioned above were reconstructed based on BI and ML phylogenetic analyses of these alignments, performed via CIPRES Science Gateway 3.1 (Miller et al. 2010). The 45S and *rpb*1 alignments were divided into five and nine partitions, respectively (45S into 18S, ITS1, 5.8S, ITS2, and 28S; *rpb*1 into five exons and four introns). In both BI and ML analyses, GTR+I+G was used as the nucleotide substitution model for each nucleotide partition, as suggested by Abadi et al. (2019).

The BI reconstructions were made based on four Markov chains run over 1 million generations in MrBayes 3.2 (Ronquist et al. 2012), sampling every 1,000 generations, with a burn-in at 30% sampled trees. The ML phylogenetic tree inferences were performed with RAxML-NG 1.0.1 (Kozlov et al. 2019), using a maximum likelihood/1000 bootstrapping run and ML-estimated proportion of invariable sites and base frequencies. The alignments and tree files were deposited as supplementary materials. Clade and node supports were considered strong, moderate, and marginal when BI and ML support values were 0.98–0.99 and 81–99%, 0.96–0.97 and 71–80%, and 0.95 and 70%, respectively. The phylogenetic trees were visualized and edited in FigTree v. 1.4.4 (http://tree.bio.ed.ac.uk/software/figtree/) and MEGA6 (Tamura et al. 2013).

To detect possible other findings of Isolate 527 and Isolate 530, their 45S sequences were used as queries in BLASTn to retrieve environmental sequences of potentially identical species from GenBank and EUKARYOME (Tedersoo et al. 2024b). The sequences were selected according to a percentage identity of > 96%. Their likely identity was then verified in BI and ML analyses of the alignments with 45SR+environmental and 45SD+environmental sequences.

## Results

### General data and phylogeny

The alignments analyzed contained 13 and six newly obtained sequences of the 45S region and the *rpb*1 gene, respectively. The numbers of analyzed sequences and species/isolates, as well as the numbers of base pairs and variable and parsimony-informative sites of each of the alignments, are presented in Table 1.

**Table 1.** Characteristics of the sequence alignments analyzed.

Name of alignment	No. of sequences	No. of fungal species	No. of base pairs	No. of variable sites	No. of parsimony informative sites
45S	167	57	2008	1104	955
45SR	66	20	1663	616	509
45SD	45	17	1763	665	582
*rpb*1R	32	11	2455	476	315
*rpb*1D	43	16	2520	532	477
45SR+*rpb*1R	66	20	4118	1081	817
45SD+*rpb*1D	45	17	4283	1183	1057

The similarities of the 45S Isolate 527 and Isolate 530 sequences used were 99.4% and 98.0%, respectively. The *rpb*1 sequences of these isolates differed by 0.3% and 0.5–0.8%, respectively.

The topologies of the trees with 45S and 45S+*rpb*1 sequences generated in the BI and ML analyses were identical (Figs 1, 2; Suppl. materials 1–3). Small and insignificant differences in the topologies of the *rpb*1 trees compared to the topologies of the 45S and 45S+*rpb*1 trees resulted from the lack of *rpb*1 sequences of nine of the 20 species and one of the 17 species shown in the 45SR+*rpb*1R and 45SD+*rpb*1D trees, respectively (Figs 1, 2; Suppl. materials 4, 5).

In the 45SR+*rpb*1R tree, Isolate 527 was placed in an autonomous clade in a basal position to the *Rhizoglomus* clade, in which *R. invermaium* and *R. melanum* were placed next to Isolate 527 (Fig. 1). The Isolate 527 clade obtained full BI and ML supports.

In the 45SD+*rpb*1D tree, Isolate 530 populated a clade sister to a clade with *D. aurea* (Fig. 2). The Isolate 530 clade obtained full and strong BI and ML support, respectively. The BI and ML supports of the *D. aurea* clade and the node connecting this clade with the Isolate 530 clade were full.

The genetic distances between the 45S sequences of Isolate 527 and its closest relatives, i.e., (i) *R. invermaium* and *R. melanum*, (ii) *Sc. sinuosa*, and (iii) *Si. neocaledonica* (Fig. 1; Suppl. material 7: table SS1) were (i) 12.4–13.2%, (ii) 15.4–17.1%, and (iii) 15.6–17.5%, respectively. In terms of the *rpb*1 sequences, exon 4 + intron 4 + exon 5 of Isolate 527 differed from those of *Sc. sinuosa* and *Si. neocaledonica* by 11.3–11.4% and 9.2–9.5%, respectively. The differences between exon 4 + exon 5 in the same comparisons were 5.9–6.0% and 6.2–6.4%, respectively.

The 45S sequences of Isolate 530 and *D. aurea* differed by 2.4–4.2% (Suppl. material 7: table S2). When the 45S sequences of all other *Dominikia* species were compared, these differences ranged from 5.0% (*D. duoreactiva* vs. *D. lithuanica*) to 13.9% (*D. iranica* vs. *D. paraminuta*). Comparisons of the entire *rpb*1 sequences and their components separately showed that (i) *D. tatrensis* and *D. aurea* differed by ≥ 1.1% in the sequence contents of intron 1 (by 6.1%), exon 3 (1.4–4.7%), and exons 1–5 plus introns 1–4 (2.4–2.8%); (ii) only the contents of intron 1, exon 3, and exons 1–5 plus introns 1–4 diverged from all compared *Dominikia* species by > 1.1% (by 6.1–22.4%, 1.4–6.0%, and 2.4–9.9%, respectively), with the exception of *D. disticha* versus *D. iranica*, *D. achra* vs. *D. duoreactiva*, and *D. achra* vs. *D. lithuanica*, for which these divergences were 1.0–1.7%; and (iii) the genetic differences between the contents of exon 5, exon 4 plus exon 5, and exons 1–5 of *D. achra* vs. *D. duoreactiva* and *D. lithuanica*, *D. aurea* vs. *D. tatrensis*, and *D. duoreactiva* vs. *D. lithuanica* were 0.2–1.1%.

### Taxonomy

**Figure 3.**
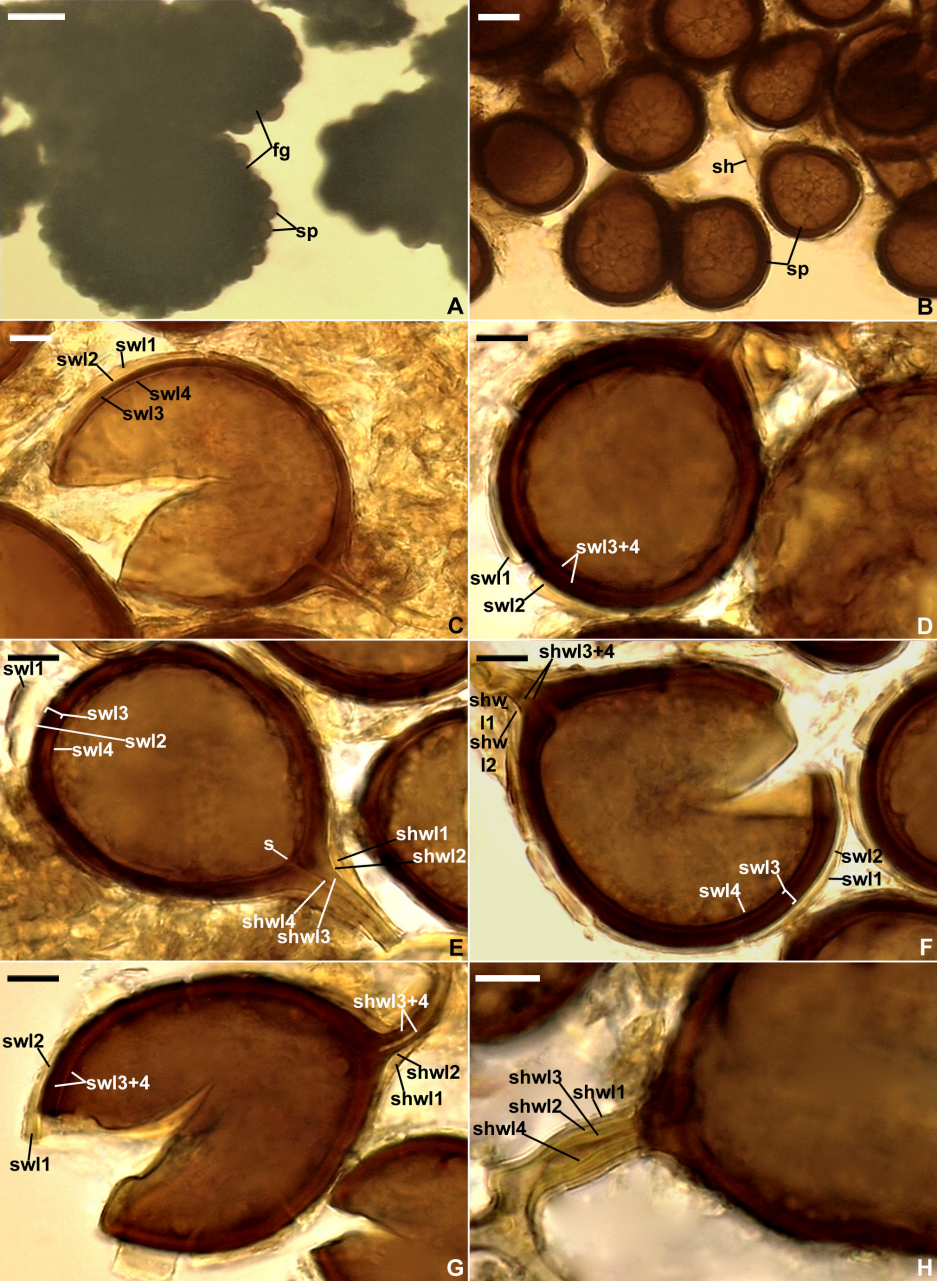
*Durabilispora carpatica*. **A**. Fused glomerocarps (fg) with spores (sp); **B**. Cluster of spores (sp) with subtending hypha (sh); **C, D**. Spore wall layers (swl) 1–4; **E–G**. Spore wall layers (swl) 1–4 continuous with subtending hyphal wall layers (shwl) 1–4; the septum (s) closing the space between the subtending hyphal lumen and spore interior is indicated; **H**. Subtending hyphal wall layers (shwl) 1–4; **A**. Glomerocarps in lactic acid; **B, D–F**. Spores in PVLG; **C, G**. Spores in PVLG+Melzer’s reagent; **A**. Light microscopy; **B–H**. Differential interference microscopy. Scale bars: 100 μm (**A**); 20 μm (**B**); 10 μm (**C–H**).

**Figure 4.**
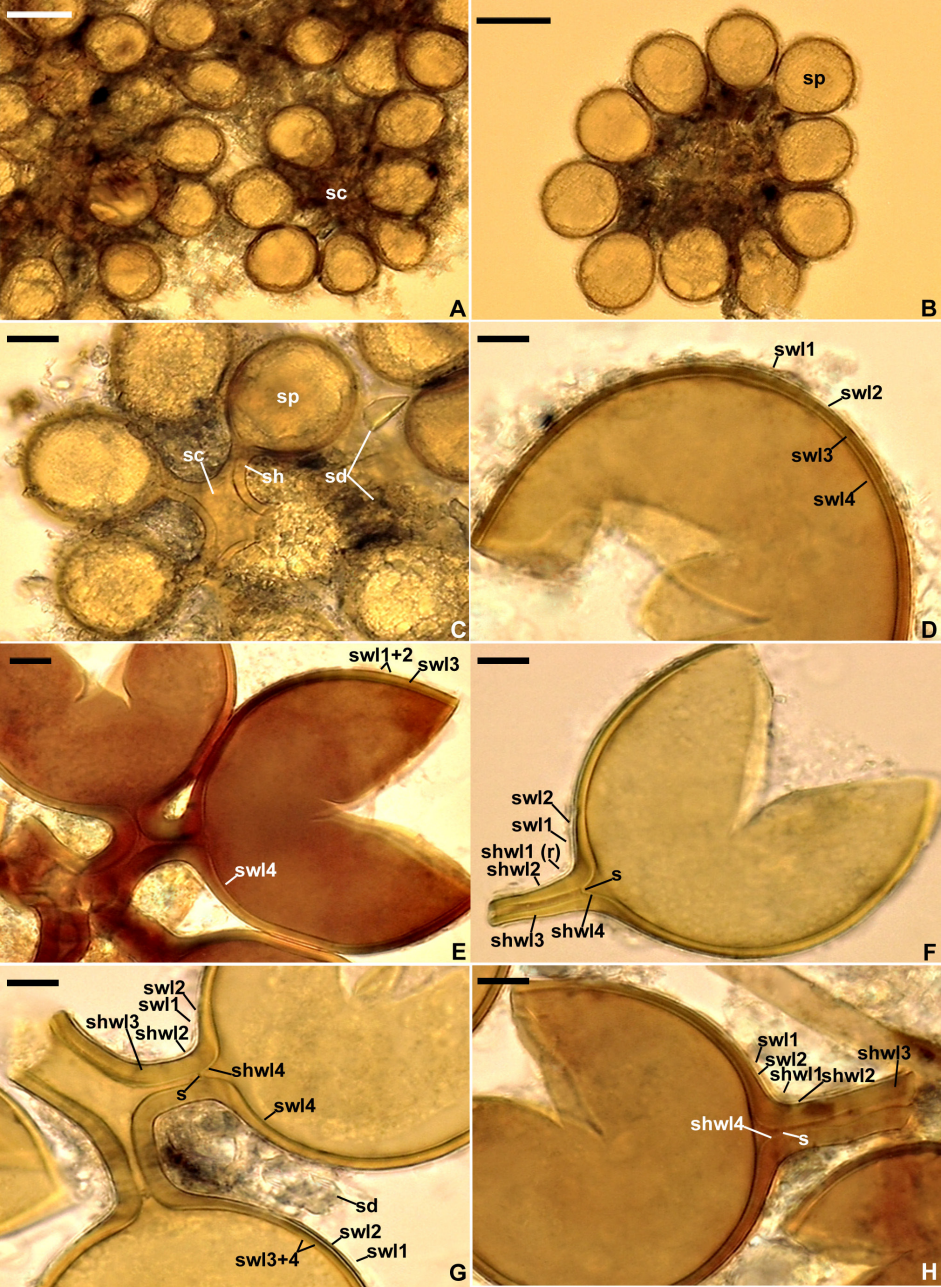
*Dominikia tatrensis*. **A**. Fused clusters with spores; one single cluster (sc) is indicated; **B**. Single cluster with spores (sp); **C**. Radially arranged spores (sp) with subtending hyphae (sh) branched from a centrally positioned thick-walled swollen cell (sc); soil debris (sd) between spores is indicated; **D, E**. Spore wall layers (swl) 1–4; **F–H**. Spore wall layers (swl) 1–4 continuous with subtending hyphal wall layers (shwl) 1–4; r in (F) = remnants of shwl 4; septum (s) separating the space between the subtending hyphal lumen and spore interior is indicated; **A, B, C, F, G**. Spores in PVLG; **D, E, H**. Spores in PVLG+Melzer’s reagent; **A–H**. Differential interference microscopy. Scale bars: 50 μm (**A**, **B**); 20 μm (**C**); 10 μm (**D–H**).

The phylogenetic analyses and sequence comparisons described above indicated that (i) Isolate 527 is an undescribed species that should represent a new genus in the family *Rhizoglomeraceae* and (ii) Isolate 530 is a new *Dominikia* species in *Dominikiaceae*. Consequently, (i) based on Isolate 527, a new genus, named *Durabilispora* gen. nov., with *Du. carpatica* sp. nov., was established, and (ii) Isolate 530 was described as *D. tatrensis* sp. nov. (Figs 1, 2, 3, 4; Suppl. materials 1–5).

#### Descriptions of a new genus and a new species

##### Durabilispora

Taxon classificationFungiGlomeralesGlomeraceae

Błaszk., Niezgoda & B.T.Goto gen. nov.

03A5EDDC-75A1-5A12-AC4E-F985EBD12F11

863195

Figs 1, 3; Suppl. materials 1, 2, 4

###### Etymology.

*Durabilispora*, referring to the durability of the components of the spore and subtending hyphal walls of the type species of this new genus.

###### Type species.

*Durabilispora carpatica* Błaszk., Zubek, Niezgoda & B.T.Goto

###### Diagnosis.

Differs from other genera of *Glomerales* (i) by forming fused glomerocarps with spores not arising radially around a central plexus of hyphae, (ii) in the structure and phenotypic properties of components of the spore and subtending hyphal walls, and (iii) in nucleotide composition of sequences of the 45S nuc rDNA region and the *rpb*1 gene (see “General data and phylogeny” and “Discussion” for details).

###### Genus description.

As that of *Durabilispora carpatica* (see below).

##### Durabilispora carpatica

Taxon classificationFungiGlomeralesGlomeraceae

Błaszk., Zubek, Niezgoda & B.T.Goto sp. nov.

79E1939E-59C3-5E60-88D8-561483942995

863196

Fig. 3A–H; Suppl. materials 1, 2, 4

###### Typification.

Poland • Małopolskie Voivodeship, glomerocarps from a trap pot culture inoculated with rhizosphere soil and root fragments of plants growing in the Tatra 1/4op site located in the lower part of the Miętusia Valley, at 938 m asl (49°5'49.25"N, 19°52'17.33"E), 18 July 2023, A. Ronikier, P. Janik (***holotype***: slide with spores ZT Myc 0067490; ***isotypes***: slides with spores nos. 4005–4011, LPPDSE). GenBank: 45S: PX641487–PX641493; *rpb*1: PX570053, PX570054.

###### Other isolates examined.

Poland • Pomeranian Voivodeship, glomerocarps from a field rhizosphere soil sample collected under *Rosa rugosa* growing in dunes of the Baltic Sea near Jastarnia (49°5'49.25"N, 19°52'17.33"E), 5 September 2015, J. Błaszkowski, slides with spores, LPPDSE.

###### Etymology.

*carpatica*, referring to the name of the mountain range, the Carpathians, in which the Tatra Mountains are located, where the holotype was collected.

###### Diagnosis.

Differs from *R. invermaium* and *R. melanum*, which were placed next to the *D. carpatica* clade (Fig. 1; Suppl. materials 1, 2, 4) in (i) the ability to form and the compactness of glomerocarps, (ii) size of spores, (iii) the spore wall structure and phenotypic features of spore wall layer 1, forming the spore surface, (iv) morphometric characters of the spore subtending hypha, (v) the origination of the septum occluding the space between the lumen of the subtending hyphal wall and the spore interior, when the septum is formed, and (vi) nucleotide composition of sequences of the 45S nuc rDNA region (see “Discussion” for details).

###### Description.

Forming compact hypogeous single and fused glomerocarps without a peridium; each glomerocarp globose to subglobose, 394–446 µm diam, to ovoid, 384–395 × 596–646 µm, with tens to approximately a hundred randomly distributed glomerospores (= spores); fused glomerocarps with two to three single glomerocarps connected by their intraglome­rocarpic hyphae (Fig. 3A, B). ***Spores*** glomoid, arising blastically at tips of subtending hyphae; orange (5B8) to yellowish brown (5E8); globose to subglobose; (35–)45(–56) µm diam; frequently ovoid to oblong; 19–57 × 24–71 µm; with one subtending hypha (Fig. 3B–H). ***Spore wall*** composed of four permanent, smooth layers (layers 1–4; Fig. 3C–G). Layer 1, forming the spore surface, uniform (without visible sublayers), semi-flexible, yellowish white (4A2) to pale yellow (4A3), (1.0–)2.4(–4.0) µm thick, tightly adherent to layer 2 (Fig. 3C–G). Layer 2 uniform, semi-flexible, hyaline to yellowish white (4A2), (0.6–)1.3(–2.0) µm thick, tightly adherent to layer 3 (Fig. 3C–G). Layer 3 laminate, semi-flexible, orange (5B8) to yellowish brown (5E8), (1.8–)3.4(–5.0) µm thick, consisting of very thin, < 0.5 µm, sublayers tightly adherent to and not separating from each other even in vigorously crushed spores (Fig. 3C–G). Layer 4 uniform, flexible to semi-flexible, orange (5B8) to yellowish brown (5E8), (0.8–)0.9(–1.0) µm thick, rarely and only slightly separating from the lower surface of layer 3 and, therefore, occasionally difficult to detect (Fig. 3C–G). Layers 1–4 do not stain in Melzer’s reagent (Fig. 3C, G). ***Subtending hypha*** pale yellow (4A3) to yellowish brown (5E8); straight or recurved, cylindrical to funnel-shaped, (9.3–)14.7(–18.8) µm wide at the spore base (Fig. 3B, C, E–H). ***Wall of subtending hypha*** pale yellow (4A3) to yellowish brown (5E8); (4.8–)7.7(–10.2) µm thick at the spore base; composed of four layers continu­ous with spore wall layers 1–4 (Fig. 3E–G, H). ***Pore*** (0.8–)1.0(–1.6) µm diam, usually open (Fig. 3C, D, F–H), very rarely occluded by a curved septum continuous with spore wall layer 4; septum 0.5–0.8 µm thick, located at the level of the inner surface of spore wall layer 3 (Fig. 3E). ***Germination*** unknown.

###### Ecology and distribution.

In the field, *Du. carpatica* most likely form mycorrhizal symbiosis with plants of the Tatra 1/4op site (Suppl. material 6) and *R. rugosa*. However, no molecular analysis was performed to confirm the supposition. Using BLASTn, GenBank, and EUKARYOME searches revealed many environmental sequences with > 96% identity to sequences of *Du. carpatica*. However, verifying BI and ML analyses with the sequences of the tree illustrated in Fig. 1 and these environmental sequences showed that only four environmental sequences, with 96.6–99.5% identity, clustered with *Du. carpatica* sequences (data not shown). All of these sequences were from forest soils collected in Estonia (accession numbers EUK1632882, EUK1634605), Mexico (EUK1683277), and Qatar (EUK1683239). Thus, *Du. carpatica* is a species that is widespread through the world, but its occurrence is rare.

##### Dominikia tatrensis

Taxon classificationFungiGlomeralesGlomeraceae

Błaszk., Zubek, Niezgoda & B.T.Goto sp. nov.

DF9C85D1-29BF-5ECA-B0DD-078F24B5D08C

863197

Fig. 4A–H; Suppl. materials 1, 3, 5

###### Typification.

Poland • Małopolskie Voivodeship, glomerocarps from a trap pot culture inoculated with rhizosphere soil and root fragments of plants growing in the Tatra 2/1op site located in the lower part of the northwestern part of the Polana Upław meadow, 1298 m asl (49°15'3.6"N, 19°52'46.92"E), 11 May 2023, A. Ronikier and P. Janik (***holotype***: slide with spores ZT Myc 0067491; ***isotypes***: slides with spores nos. 4012–4017, LPPDSE). GenBank: 45S: PX641494–PX641499; *rpb*1: PX570049–PX570052.

###### Etymology.

Latin, *tatrensis*, referring to the Tatra Mountains, where specimens of this species were originally found.

###### Diagnosis.

Differs from (A) *D. aurea*, the closest phylogenetic relative (Fig. 2; Suppl. materials 1, 3, 5) in (i) the organization of spores in glomerocarps, (ii) the spore wall structure, (iii) the durability of spore wall layer 1, forming the spore surface, (iii) morphometric features of the spore wall, subtending hypha and its pore, (iv) the origination of the septum occluding the pore, and (v) nucleotide composition of sequences of the 45S nuc rDNA region and the *rpb*1 gene, and (B) *Glomus fuegianum*, the morphologically most similar species, in the formation of glomerocarps lacking peridium and glebal hyphae, as well as in the spore wall structure (see “General data and phylogeny” and “Discussion” for details).

###### Description.

Forming compact hypogeous single and fused glomerocarps without a peridium; each glomerocarp globose to subglobose, 154–280 µm diam, to ovoid, 166–190 × 213–291 µm, with five to twelve radially arranged glomerospores (= spores); fused glomerocarps with two to six single glomerocarps; each glomerocarp probably formed from a swollen cell (cha­racterized below) produced at the tip of a hypha branched from a parent hypha continuous with an extraradical mycorrhizal hypha (Fig. 4A–C). ***Spores*** glomoid, arising blastically at tips of subtending hyphae branched radially from a centrally located swollen cell, 22.3 × 28.0 µm wide with a wall 3.9–5.6 µm thick (Fig. A, C). ***Spores*** greyish yellow (4B4–4B6); globose to subglobose; (48–)58(–61) µm diam; rarely ovoid; 42–60 × 52–66 µm; with one subtending hypha (Fig. 4A–H). ***Spore wall*** composed of four layers (layers 1–4), including three permanent, smooth layers (layers 2–4; Fig. 4D–H). Layer 1, forming the spore surface, mucilaginous, flexible to semi-flexible, hyaline, (0.8–)1.0(–1.6) µm thick when intact, slowly deteriorating with age, usually present as a highly decomposed structure even in older spores (Fig. 4D–H). Layer 2 uniform (without visible sublayers), semi-flexible, hyaline, (0.8–)1.5(–2.2) µm thick, tightly adherent to layer 3 (Fig. 4D–H). Layer 3 laminate, semi-flexible, greyish yellow (4B4–4B6), (1.8–)2.3(–3.0) µm thick, consisting of very thin, < 0.5 µm, sublayers tightly adherent to and not separating from each other even in vigorously crushed spores (Fig. 4D, E, G). Layer 4 uniform, semi-flexible, greyish yellow (4B4–4B6), (0.6–)0.8(–1.2) µm thick, usually rarely and only slightly separating from the lower surface of layer 3, usually easy to detect (Fig. 4D, E, G). Only layer 1 stains pale red (7B3) to light brown (7D6) in Melzer’s reagent (Fig. 4D, E, H). ***Subten­ding hypha*** greyish yellow (4B4–4B6); straight or recurved, cylindrical, rarely funnel-shaped, (11.2–)14.6(–18.8) µm wide at the spore base (Fig. 4C, E–H). ***Wall of subtending hypha*** greyish yellow (4B4–4B6); (3.4–)6.5(–8.6) µm thick at the spore base; composed of four layers continuous with spore wall layers 1–4 (Fig. 4F, G, H). ***Pore*** (0.8–)1.4(–2.0) µm diam, usually occluded by a curved septum continuous with spore wall layer 4; septum 0.8–1.0 µm thick, located at or 3.5–4.0 µm below the spore base (Fig. 4F–H). ***Germination*** unknown.

###### Ecology and distribution.

In the field, *D. tatrensis* probably formed arbuscular mycorrhizal symbiosis with plant(s) of the Tatra 2/1op site (Suppl. material 6). No molecular analysis was performed to confirm the existence of the symbiosis. BLASTn queries sent to GenBank and EUKARYOME showed many environmental sequences with > 96% identity to sequences of the new species. However, BI and ML analyses of the 45SD alignment extended by the environmental sequences showed that only five of them clustered with *D. tatrensis* sequences (data not shown), suggesting their origin from the new species. The sequences were EUK1633193, EUL1631679, HE775327, HE775330, and JX096576 with 97.8%, 98.3%, 98.39%, 98.52%, and 96.55% identities, respectively. The sequences EUK1633193 and EUL1631679 were obtained from Estonian temperate shrubland and temperate broadleaf forest soils, respectively. The HE775327 and HE775330 sequences were obtained from roots of *Brachypodium pinnatum* (L.) P. Beauv. growing in a meadow of North Bohemia, Czech Republic. The JX096576 sequence was obtained from a soil sample collected from the Qinghai-Tibet Plateau, China. These data suggest that *D. tatrensis* has a widespread global distribution, although it appears to occur rarely.

## Discussion

The phylogenetic analyses performed as part of this study confirmed the initial hypotheses, based on sequence comparisons and morphological observations, that two fungi producing glomoid spores in glomerocarps, preliminarily named Isolate 527 (= Isolate 299) and Isolate 530, are new taxa of the order *Glomerales* in the phylum *Glomeromycota*. These analyses indicated that Isolate 527 represents a new genus in the family *Rhizoglomeraceae*, here described as *Durabilispora* with *Du. carpatica* sp. nov., and Isolate 530 is a new *Dominikia* species in the family *Dominikiaceae*, which is described as *D. tatrensis* (Figs 1, 2; Suppl. materials 1–5).

Five facts convincingly supported the statements presented above. (1) In all trees, the fully or highly supported *Du. carpatica* clade showed the same level of dichotomy as, e.g., the generic clades *Rhizoglomus*, *Sclerocystis*, and *Silvaspora* (Fig. 1; Suppl. materials 1, 2). (2) In the EUKARYOME database, the sequences EUK1632882, EUK1634605, EUK1683277, and EUK1683239, listed in “Ecology and distribution” for *Du. carpatica*, were assigned to an undescribed generic clade called gen21 (Tedersoo et al. 2024b), which is described here as *Durabilispora*. In the tree by Tedersoo et al. (2024a), the gen21 clade was placed next to the *Rhizoglomus* clade, as in the trees in this study. (3) The 45S sequence divergences between *Du. carpatica* versus *R. invermaium* (12.7–13.2%) and *R. melanum* (12.4–13.0%), which populated clades located next to each other, were close to the divergences between *Du. carpatica* and *Sc. sinuosa* (13.4–17.1%) and *Du. carpatica* and *Si. neocaledonica* (15.6–17.5%), species of the two next genera closest to *Durabilispora* in all trees with 45S sequences (Fig. 1; Suppl. materials 1, 2); the divergences clearly exceeded the 10% 45S sequence threshold that delimits other generic clades in *Glomerales* (da Silva et al. 2024). The same relationships were found between the divergences of the *Du. carpatica rpb*1 sequences and those of (i) the most closely related *Rhizoglomus* species for which *rpb*1 sequences are available, as well as (ii) *Si. neocaledonica* and *Sc. sinuosa* (Fig. 1; Suppl. materials 1, 2, 4). These divergences, which were 5.5–9.9%, 6.2–9.3%, and 5.9–11.4%, respectively, were comparable to those between, e.g., *Complexispora* and *Glomus* (9.7–9.9%), the sister genera in the trees depicted in Fig. 2, Suppl. materials 1, 3, and the sister generic clades represented by, e.g., *Oehlia diaphana* and *Sc. sinuosa* (10.3–10.4%), as well as *Funneliformis mosseae* and *Blaszkowskia deserticola* (11.4%) (Stockinger et al. 2014). It is noteworthy that the taxonomic resolution of *rpb*1 sequences is much higher than that of 45S sequences because the *rpb*1 gene has several variable regions useful for species discrimination and is a single-copy gene in fungi, avoiding problems with paralogs (Stockinger et al. 2014). This, in turn, makes the *rpb*1 phylogenies relatively more reliable (Stockinger et al. 2014). Importantly, similarly to the present study, no conflict was found between the phylogenies reconstructed from analyses of 45S, *rpb*1, and 45S+*rpb*1 sequences, which were performed in the last 8 years and published in 15 articles (Błaszkowski et al. 2025). (4) (i) All phylogenetic analyses placed *D. tatrensis* in a separate species clade sister to the *D. aurea* clade (Fig. 2; Suppl. materials 1, 3, 5), and (ii) the sequence divergences, especially those regarding *rpb*1 sequences obtained following the use of primers designed by Stockinger et al. (2014), between these two species (see “General data and phylogeny”) clearly exceeded the widely accepted thresholds of conspecificity, i.e., 97% and ca. 99.0% for 18S-ITS-28S and *rpb*1 sequences, respectively (Stockinger et al. 2014; Corazon-Guivin et al. 2019a, 2019b). (5) *Du. carpatica* and *D. tatrensis* differ clearly in morphology from their closest relatives and other members of *Glomerales*.

In addition to *Du. carpatica*, the only other *Glomeromycota* species described to form fused glomerocarps is *Sclerocystis coremioides* (Almeida and Schenck 1990; Goto et al. 2016). However, spores in the glomerocarps of this species are regularly arranged because they arise radially around a central plexus of hyphae. Furthermore, the glomerocarps of *S. coremioides* are covered by a peridium. In the glomerocarps of *Du. carpatica*, spores are randomly distributed because they develop from hyphae branching off from other hyphae located at various locations within the glomerocarps, which, moreover, are not covered by a peridium.

The main morphological differences between *Du. carpatica* and *R. invermaium* reside in the spore wall structure and the phenotypic features of spore wall layer 1, forming the spore surface. The spore wall of *Du. carpatica* includes four layers, while that of *R. invermaium* consists of two layers (Hall 1977; Błaszkowski 2012), lacking spore wall layers 2 and 4 of the new species (Fig. 3C–G). Spore wall layer 1 of *Du. carpatica* is yellowish white (4A2) to pale yellow (4A3) (Fig. 3C–G) (vs. colorless in *R. invermaium*) and up to 2.7-fold thicker. At the spore base, the subtending hypha and its wall in *Du. carpatica* are 1.2–1.6-fold and 1.2–1.9-fold wider and thicker, respectively. In addition, while the subtending hyphal pore of *Du. carpatica* is usually open (Fig. 3C, D), rarely occluded by a septum continuous with spore wall layer 4 (Fig. 3E), that of *R. invermaium* is closed by a septum continuous with some innermost laminae of spore wall layer 2. Finally, the *Du. carpatica* glomerocarps contain tightly packed spores and are, therefore, compact (Fig. 3A, B), whereas the *R. invermaium* glomerocarps consist of loosely distributed spores.

Numerous morphological features also clearly separate *Du. carpatica* from *R. melanum*. The spore wall of the latter species does not have spore wall layer 4 of the former species (Fig. 3C–G; Sudová et al. 2015). Spore wall layer 1 of *Du. carpatica* is permanent, always present even in old specimens (Fig. 3C–G; vs. it is impermanent, usually completely sloughed off in newly mature spores in *R. melanum*), colored (vs. colorless), and 1.3–3.3-fold thicker. Spore wall layer 2 of *Du. carpatica* is also permanent (Fig. 3C–G), while that of *R. melanum* is evanescent to semi-persistent, despite being much, 2.6–4.7-fold, thicker than spore wall layer 2 of the new species. Importantly, in *R. melanum*, this layer is brittle and splits into irregular pieces upon crushing of spores (vs. retains its integrity even after vigorous crushing of spores in *Du. carpatica*; Fig. 3C–G). The laminate spore wall layer 3 of *Du. carpatica* is 2.4–4.0-fold thinner than the laminate spore wall layer 3 of *R. melanum*. Instead, at the spore base, the wall of the subtending hypha and its pore in *Du. carpatica*, which occasionally is occluded (Fig. 3E; vs. open in *R. melanum*), are 2.1–3.2-fold and 4.3–4.7-fold thicker and narrower, respectively. Finally, while *Du. carpatica* spores are produced only in compact glomerocarps (Fig. 3A, B), those of *R. melanum* are produced only singly in the soil.

*Dominikia tatrensis* and *D. aurea* differ mainly in the organization of spores in glomerocarps, the composition of the spore wall, and the durability of spore wall layer 1. *Dominikia tatrensis* glomerocarps are distinguished by radially arranged spores around a centrally located swollen cell giving rise to subtending hyphae (Fig. 4A–C). In *D. aurea* glomerocarps, however, spores are randomly embedded between interwoven hyphae and amorphous material (Oehl et al. 2003; Błaszkowski 2012), which are absent in *D. tatrensis* glomerocarps. The *D. tatrensis* spore wall consists of four layers (Fig. 4D, E, G), including layers 2 and 4, which are not present in the *D. aurea* spore wall. Spore wall layer 1 of *D. tatrensis* decomposes slowly and is generally present as a highly deteriorated structure in mature and older spores (Fig. 4D–H); thus, it is more durable than that of *D. aurea*, which is usually completely sloughed off in mature spores. In addition, the spore wall of *D. tatrensis* is 1.1–1.5-fold thicker; at the spore base, the spore subtending hypha is ca. 1.9-fold wider, has a 1.1–1.7-fold thicker wall, its pore is up to 1.3-fold wider, and it is closed by a septum continuous with spore wall layer 4 (Fig. 4F, G, H; vs. occluded by some innermost laminae of the laminate spore wall layer 2 in *D. aurea*).

Of the non-sequenced *Glomeromycota* species, *D. tatrensis* is most similar in morphology to *Glomus fuegianum*, characterized by Błaszkowski et al. (1998), Pegler et al. (1993), Rosemary and Godfrey (1957), Thaxter (1922), and Goto and Maia (2005); Błaszkowski et al. (1998) examined specimens collected in Poland and those borrowed from the herbarium of the Royal Botanic Gardens at Kew, UK. Both species form glomerocarps consisting of spore groups, and in each group, spores develop radially from a centrally located inflated cell. Three main characters clearly separate the two species. The glomerocarps of *G. fuegianum* are covered by thin-walled interwoven hyphae, forming a peridium that is not produced by *D. tatrensis*. Within these *G. fuegianum* glomerocarps, the spore groups are separated by distinct strands of compact parallel hyphae, which are absent in *D. tatrensis* glomerocarps. The spore wall of *D. tatrensis* consists of four layers, whereas that of *G. fuegianum* was described to be one- (Thaxter 1922), two- (Rosemary and Godfrey 1957; Błaszkowski et al. 1998; Goto and Maia 2005), and three-layered (Pegler et al. 1993).

*Parvocarpum badium* and *Si. hoi* were not included in the phylogenetic analyses because the available sequences of these species were either relatively very short, aligned improperly, or came only from the 18S gene. In the case of *P. badium*, only approximately 300 base pairs of the 18S-ITS1 sequences aligned with other sequences used in the analyses and, moreover, part of the highly variable ITS1 region aligned with 28S gene sequences (data not shown). *Simiglomus hoi* has been molecularly characterized only from the 18S gene, whose content has low taxonomic resolution for *Glomeromycota* members and, in the alignments, was represented by ca. 240 base pairs. Thus, this decision is consistent with the conclusions of, e.g., da Silva et al. (2024) and Redecker et al. (2013) that the phylogenetic informativeness of such sequences is insufficient to reconstruct reliable phylogenetic positions of AMF. Importantly, in the tree by Tedersoo et al. (2024b), the generic clade with *P. badium* and numerous environmental sequences is two generic clades away from the generic clade named gen21, which contains numerous environmental sequences (see also Tedersoo et al. 2024a), including those that clustered with *Du. carpatica* sequences in the verifying analyses (see “Ecology and distribution” for *Du. carpatica*). Finally, these two species differ clearly in terms of morphology from the four main species discussed in this paper.

*Parvocarpum badium* produces spores in compact glomerocarps, as do *Du. carpatica* and *D. tatrensis* (Figs 3A, 3B, 4A–C; Oehl et al. 2005; Błaszkowski 2012). Furthermore, the spores of *P. badium* are arranged radially in these glomerocarps, as are spores in the *D. tatrensis* glomerocarps (Fig. 4A–C). However, *P. badium* spores arise from a centrally located plexus of hyphae, while *D. tatrensis* spores develop from a centrally located inflated cell (Fig. 4A, C). In *Du. carpatica* glomerocarps, the spores are randomly arranged (Fig. 3B). In addition, *Du. carpatica*, *D. tatrensis*, *R. invermaium*, and *R. melanum* differ clearly from *P. badium* and *Si. hoi* with respect to most of the following taxonomically significant morphological characters: spore color, morphometric characters of spores and spore subtending hyphae, spore wall structure, phenotypic properties of spore wall components, and the presence of a septum closing the spore subtending hyphal pore (Hall 1977; Berch and Trappe 1985; Oehl et al. 2011; Błaszkowski 2012; Goto et al. 2013; Sudová et al. 2015; Goto et al. 2025).

Finally, the analyses showed that, in contrast to the genetic contents of intron 1, exon 3, and exons 1–5 plus introns 1–4, using only exon 4 plus exon 5 sequences was insufficient to separate *D. aurea* from *D. tatrensis*, as well as *D. achra* from *D. duoreactiva* and *D. duoreactiva* from *D. lithuanica* (Suppl. material 7) based on the distance threshold level (~1.1%) proposed by Stefani et al. (2025). The same was also true for species of other *Glomeromycota* taxa, which, similarly to the mentioned *Dominikia* species, differ clearly in terms of 45S phylogeny and morphology (Suppl. material 7; Berch and Fortin 1983, 1984; Błaszkowski et al. 2009, 2016, 2021b, 2024; Błaszkowski 2012; unpubl. data). For example, comparisons of the contents of (i) exon 3 equated *Glomus atlanticum* with *G. rugosae* and (ii) exon 3, exons 4 plus 5, and exons 1–5 suggested species identity in comparisons of *G. atlanticum* vs. *G. macrocarpum*, *G. atlanticum* vs. *G. rugosae*, *G. bareae* vs. *G. tetrastratosum*, and *G. macrocarpum* vs. *G. rugosae*. In contrast, using the sequences of exons 1–5 plus introns 1–4, as well as the sequences of the 45S segment, clearly separated all these species. The differences between these species were 1.6–2.9% and 3.4–5.9% for the *rpb*1 and 45S sequences, respectively. Therefore, in cases of such conflicts, the full *rpb*1 sequences sensuStockinger et al. (2014) are recommended.

## Supplementary Material

XML Treatment for Durabilispora

XML Treatment for Durabilispora carpatica

XML Treatment for Dominikia tatrensis

## Additional information

### Artificial Intelligence (AI) use

The authors accept full responsibility for the content of the manuscript, including the disclosure of any use of AI.

No AI tools were used in the preparation of this manuscript.

### Author ORCIDs

J. Błaszkowski https://orcid.org/0000-0003-3688-164X

P. Milczarski https://orcid.org/0000-0003-2622-8938

R. Malinowski https://orcid.org/0000-0003-3413-2653

P. Niezgoda https://orcid.org/0000-0002-0560-6154

B.T. Goto https://orcid.org/0000-0001-6157-4954

A. Ronikier https://orcid.org/0000-0003-0193-8685

M. Stanek https://orcid.org/0000-0001-9858-6721

P. Janik https://orcid.org/0000-0002-4106-636X

S. Zubek https://orcid.org/0000-0003-1338-9572

### Data availability

Datasets generated during and analyzed during the current study are available from the corresponding author upon request.

## Supplementary materials

Supplementary material 1 Phylogenetic data Data type docx Explanation note A 50% majority-rule consensus tree from the Bayesian analysis of 45S nuc rDNA sequences of *Durabilispora carpatica* (Isolate 527), *Dominikia tatrensis* (Isolate 530), and members of the other genera of *Glomerales* representing the ingroup, as well as *Entrophospora claroidea* serving as the outgroup. The new genus and species are in bold font. The Bayesian posterior probabilities ≥ 0.90 and ML bootstrap values ≥ 50% are shown near the branches, respectively. Bar indicates a 0.1 expected change per site per branch. https://binary.pensoft.net/file/1695028This dataset is made available under the Open Database License (http://opendatacommons.org/licenses/odbl/1.0/). The Open Database License (ODbL) is a license agreement intended to allow users to freely share, modify, and use this Dataset while maintaining this same freedom for others, provided that the original source and author(s) are credited. Janusz Błaszkowski, Paweł Milczarski, Ryszard Malinowski, Piotr Niezgoda, Bruno Tomio Goto, Anna Ronikier, Małgorzata Stanek, Paulina Janik, Szymon Zubek

Supplementary material 2 Phylogenetic data Data type docx Explanation note A 50% majority-rule consensus tree from the Bayesian analysis of 45S nuc rDNA sequences of *Durabilispora carpatica* (Isolate 527) and 17 *Rhizoglomus* species representing the ingroup, as well as *Sclerocystis sinuosa* and *Silvaspora neocaledonica*, serving as the outgroup. The new genus and species are in bold font. The Bayesian posterior probabilities ≥ 0.90 and ML bootstrap values ≥ 50% are shown near the branches, respectively. Bar indicates a 0.03 expected change per site per branch. https://binary.pensoft.net/file/1695029This dataset is made available under the Open Database License (http://opendatacommons.org/licenses/odbl/1.0/). The Open Database License (ODbL) is a license agreement intended to allow users to freely share, modify, and use this Dataset while maintaining this same freedom for others, provided that the original source and author(s) are credited. Janusz Błaszkowski, Paweł Milczarski, Ryszard Malinowski, Piotr Niezgoda, Bruno Tomio Goto, Anna Ronikier, Małgorzata Stanek, Paulina Janik, Szymon Zubek

Supplementary material 3 Phylogenetic data Data type docx Explanation note A 50% majority-rule consensus tree from the Bayesian analysis of 45S nuc rDNA sequences of *Dominikia tatrensis* (= Isolate 530) and 13 other *Dominikia* species representing the ingroup, as well as *Complexispora multistratosa*, *Glomus macrocarpum*, and *Sclerocarpum amazonicum* serving as the outgroup. The new species is in bold font. The Bayesian posterior probabilities ≥ 0.90 and ML bootstrap values ≥ 50% are shown near the branches, respectively. Bar indicates a 0.02 expected change per site per branch. https://binary.pensoft.net/file/1695030This dataset is made available under the Open Database License (http://opendatacommons.org/licenses/odbl/1.0/). The Open Database License (ODbL) is a license agreement intended to allow users to freely share, modify, and use this Dataset while maintaining this same freedom for others, provided that the original source and author(s) are credited. Janusz Błaszkowski, Paweł Milczarski, Ryszard Malinowski, Piotr Niezgoda, Bruno Tomio Goto, Anna Ronikier, Małgorzata Stanek, Paulina Janik, Szymon Zubek

Supplementary material 4 Phylogenetic data Data type docx Explanation note A 50% majority-rule consensus tree from the Bayesian analysis of *rpb*1 sequences of *Durabilispora carpatica* (Isolate 527) and eight *Rhizoglomus* species representing the ingroup, as well as *Sclerocystis sinuosa* and *Silvaspora neocaledonica*, serving as the outgroup. The new genus and species are in bold font. The Bayesian posterior probabilities ≥ 0.90 and ML bootstrap values ≥ 50% are shown near the branches, respectively. Bar indicates a 0.2 expected change per site per branch. https://binary.pensoft.net/file/1695031This dataset is made available under the Open Database License (http://opendatacommons.org/licenses/odbl/1.0/). The Open Database License (ODbL) is a license agreement intended to allow users to freely share, modify, and use this Dataset while maintaining this same freedom for others, provided that the original source and author(s) are credited. Janusz Błaszkowski, Paweł Milczarski, Ryszard Malinowski, Piotr Niezgoda, Bruno Tomio Goto, Anna Ronikier, Małgorzata Stanek, Paulina Janik, Szymon Zubek

Supplementary material 5 Phylogenetic data Data type docx Explanation note A 50% majority-rule consensus tree from the Bayesian analysis of *rpb*1 sequences of *Dominikia tatrensis* (Isolate 530) and 11 other *Dominikia* species representing the ingroup, as well as *Complexispora multistratosa*, *Glomus macrocarpum*, and *Sclerocarpum amazonicum* serving as the outgroup. The new species is in bold font. The Bayesian posterior probabilities ≥ 0.90 and ML bootstrap values ≥ 50% are shown near the branches, respectively. Bar indicates a 0.02 expected change per site per branch. https://binary.pensoft.net/file/1695032This dataset is made available under the Open Database License (http://opendatacommons.org/licenses/odbl/1.0/). The Open Database License (ODbL) is a license agreement intended to allow users to freely share, modify, and use this Dataset while maintaining this same freedom for others, provided that the original source and author(s) are credited. Janusz Błaszkowski, Paweł Milczarski, Ryszard Malinowski, Piotr Niezgoda, Bruno Tomio Goto, Anna Ronikier, Małgorzata Stanek, Paulina Janik, Szymon Zubek

Supplementary material 6 List ot plants Data type docx Explanation note Plants inhabiting sites in which *Durabilispora carpatica* and *Dominikia tatrensis* occurred. https://binary.pensoft.net/file/1695033This dataset is made available under the Open Database License (http://opendatacommons.org/licenses/odbl/1.0/). The Open Database License (ODbL) is a license agreement intended to allow users to freely share, modify, and use this Dataset while maintaining this same freedom for others, provided that the original source and author(s) are credited. Janusz Błaszkowski, Paweł Milczarski, Ryszard Malinowski, Piotr Niezgoda, Bruno Tomio Goto, Anna Ronikier, Małgorzata Stanek, Paulina Janik, Szymon Zubek

Supplementary material 7 Phylogenetic data Data type docx Explanation note **table SS1**. Percent genetic identities between the sequences of intron 4, exon 5, and intron 4 plus exon 5 of the *rpb*1 gene and the 45S nuc rDNA region of *Durabilispora carpatica* gen. nov. et sp. nov. and representatives of the three genera most closely related to *Durabilispora*. **table S2**. Percent genetic identities between the sequences of five exons and four introns of the *rpb*1 gene and the 45S nuc rDNA region of nine *Dominikia* species. **table S3**. Percent genetic identities between the sequences of intron 1, exon 3, exons 4 and 5, exons 1–5, exons 1–5 plus introns 1–4 of the *rpb*1 gene and the 45S nuc rDNA region of five *Glomus* species. https://binary.pensoft.net/file/1695034This dataset is made available under the Open Database License (http://opendatacommons.org/licenses/odbl/1.0/). The Open Database License (ODbL) is a license agreement intended to allow users to freely share, modify, and use this Dataset while maintaining this same freedom for others, provided that the original source and author(s) are credited. Janusz Błaszkowski, Paweł Milczarski, Ryszard Malinowski, Piotr Niezgoda, Bruno Tomio Goto, Anna Ronikier, Małgorzata Stanek, Paulina Janik, Szymon Zubek

Supplementary material 8 Details of isolates Data type docx Explanation note Details of isolates included in the molecular study. https://binary.pensoft.net/file/1695035This dataset is made available under the Open Database License (http://opendatacommons.org/licenses/odbl/1.0/). The Open Database License (ODbL) is a license agreement intended to allow users to freely share, modify, and use this Dataset while maintaining this same freedom for others, provided that the original source and author(s) are credited. Janusz Błaszkowski, Paweł Milczarski, Ryszard Malinowski, Piotr Niezgoda, Bruno Tomio Goto, Anna Ronikier, Małgorzata Stanek, Paulina Janik, Szymon Zubek
