# Supplementary material for: A new genus (Durabilispora) and two new species (D. carpatica, Dominikia tatrensis) in Glomerales (Glomeromycota)
Source: MycoKeys. 2026 Jun 24;134:313–40. doi: 10.3897/mycokeys.134.187344 (PMC13324476; doi:10.3897/mycokeys.134.187344)
Supplement: Supplementary material 3 — Phylogenetic data [file mycokeys-134-313-s003.docx]

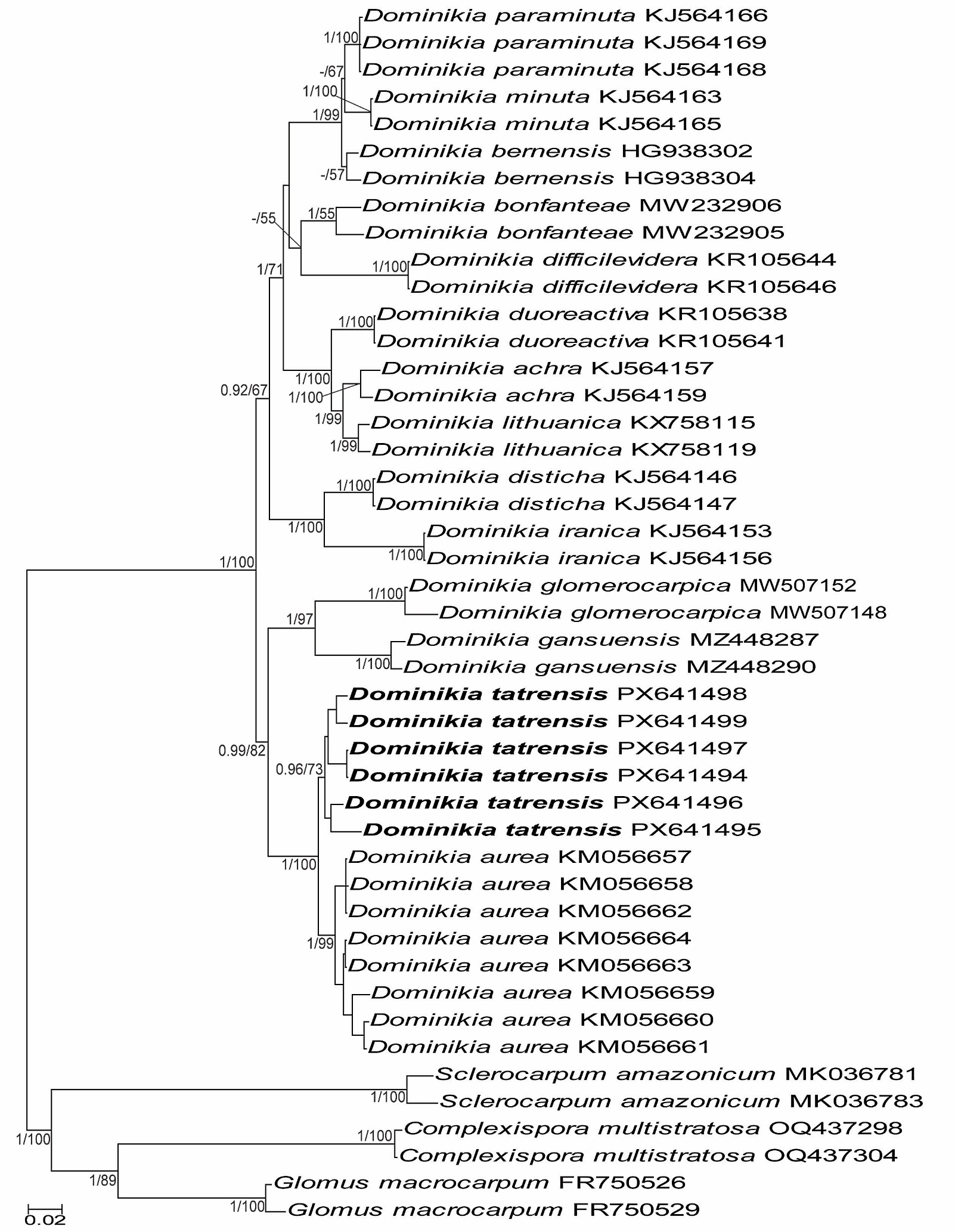


**Supplementary material 3.** 50% majority-rule consensus tree from the Bayesian analysis of 45S nuc rDNA sequences of *Dominikia tatrensis* (= Isolate 530) and 13 other *Dominikia* species representing the ingroup, as well as *Complexispora multistratosa*, *Glomus macrocarpum*, and *Sclerocarpum amazonicum* serving as outgroup. The new species is in bold font. The Bayesian posterior probabilities ≥0.90 and ML bootstrap values ≥50% are shown near the branches, respectively. Bar indicates 0.02 expected change per site per branch.
