# Supplementary material for: A new genus (Durabilispora) and two new species (D. carpatica, Dominikia tatrensis) in Glomerales (Glomeromycota)
Source: MycoKeys. 2026 Jun 24;134:313–40. doi: 10.3897/mycokeys.134.187344 (PMC13324476; doi:10.3897/mycokeys.134.187344)
Supplement: Supplementary material 4 — Phylogenetic data [file mycokeys-134-313-s004.docx]

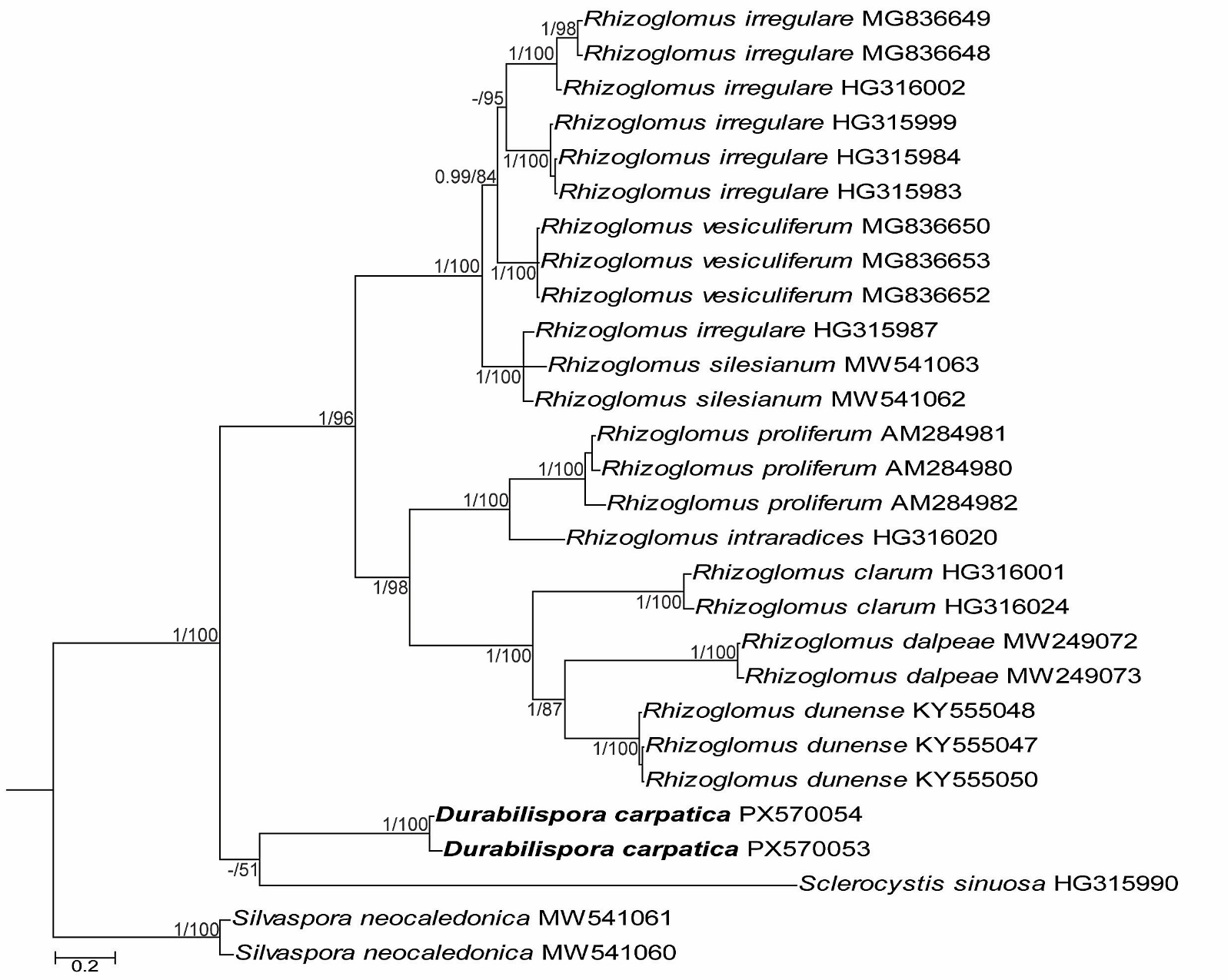


**Supplementary material 4.** 50% majority-rule consensus tree from the Bayesian analysis of sequences of *rpb1* sequences of *Durabilispora carpatica* (Isolate 527) and eight *Rhizoglomus* species representing the ingroup, as well as *Sclerocystis sinuosa* and *Silvaspora neocaledonica* serving as outgroup. The new genus and species are in bold font. The Bayesian posterior probabilities ≥0.90 and ML bootstrap values ≥50% are shown near the branches, respectively. Bar indicates 0.2 expected change per site per branch.
