# Supplementary material for: A new genus (Durabilispora) and two new species (D. carpatica, Dominikia tatrensis) in Glomerales (Glomeromycota)
Source: MycoKeys. 2026 Jun 24;134:313–40. doi: 10.3897/mycokeys.134.187344 (PMC13324476; doi:10.3897/mycokeys.134.187344)
Supplement: Supplementary material 6 — List ot plants [file mycokeys-134-313-s006.docx]

**Supplementary material 6.** Plants inhabiting sites, in which *Durabilispora carpatica* and *Dominikia tatrensis* occurred.

| AM fungal species | Site | Plants |
| --- | --- | --- |
| *Durabilispora carpatica* | Tatra 1/4op | *Agrostis gigantea* Roth, *Calamagrostis arundinacea* (L.) Roth, *Cirsium arvense* (L.) Scop., *Dactylis glomerata* L. subsp. *glomerata*, *Deschampsia flexuosa* (L.) Trin., *Epilobium montanum* L., *Festuca pratensis* Huds., *Fragaria vesca* L., *Galeobdolon luteum* Huds., *Galium aparine* L., *Galium odoratum* (L.) Scop., *Hieracium murorum* L., *Homogyne alpina* (L.) Cass., *Hypericum maculatum* Crantz, *Leontodon hispidus* L. subsp. *hastilis* (L.) Rchb., *Luzula pallescens* Sw., *Maianthemum bifolium* (L.) F. W. Schmidt, *Oxalis acetosella* L., *Phyteuma spicatum* L., *Pimpinella major* (L.) Huds., *Potentilla erecta* (L.) Raeusch., *Symphytum tuberosum* L., *Taraxacum officinale* agg., *Veronica chamaedrys* L. s. str., *Veronica officinalis* L. |
| *Dominikia tatrensis* | Tatra 2/1op | *Achillea millefolium* L. s. str., *Alchemilla* sp., *Carduus personata* (L.) Jacq., *Chaerophyllum hirsutum* L., *Galium anisophyllon* Vill., *Heracleum sphondylium* L. s. str., *Hieracium murorum* L., *Homogyne alpina* (L.) Cass., *Hypericum maculatum* Crantz, *Leontodon hispidus* L. subsp. *hispidus*, *Listera ovata* (L.) R. Br., *Malaxis monophyllos* (L.) Sw., *Pimpinella major* (L.) Huds., *Potentilla aurea* L., *Primula elatior* (L.) Hill, *Prunella vulgaris* L., *Sanicula europaea* L., *Sesleria tatrae* (Degen) Deyl, *Soldanella carpatica* Vierh., *Thymus* sp., *Trifolium spadiceum* L., *Viola biflora* L. |
