# Supplementary material for: A new genus (Durabilispora) and two new species (D. carpatica, Dominikia tatrensis) in Glomerales (Glomeromycota)
Source: MycoKeys. 2026 Jun 24;134:313–40. doi: 10.3897/mycokeys.134.187344 (PMC13324476; doi:10.3897/mycokeys.134.187344)
Supplement: Supplementary material 7 — Phylogenetic data [file mycokeys-134-313-s007.docx]

**Supplementary table S1.** Percent genetic identities between the sequences

of intron 4, exon 5, and intron 4 plus exon 5 of the *rpb1* gene, and the 45S nuc

rDNA region of *Durabilispora carpatica* gen. nov. et sp. nov. and representatives

of the three genera most closely related to *Durabilispora.*

| **Exon 4‒Intron 4‒Exon 5** | | | | |
| --- | --- | --- | --- | --- |
|  | *carpatica* | *irregulare* | *neocaledonica* | *sinuosa* |
| *carpatica* |  | 90.1-90.4 | 90.7-90.8 | 88.6-88.7 |
| *irregulare* |  |  | 89.2-89.9 | 90.1-90.4 |
| *neocaledonica* |  |  |  | 87.2-87.4 |
| **Exons 4 plus 5** | | | | |
|  | *carpatica* | *irregulare* | *neocaledonica* | *sinuosa* |
| *carpatica* |  | 94.1-94.5 | 93.6-93.8 | 94.0-94.1 |
| *irregulare* |  |  | 93.1-93.4 | 94.3-94.5 |
| *neocaledonica* |  |  |  | 92.9-93.1 |
| **45S** | | | | |
|  | *carpatica* | *irregulare* | *neocaledonica* | *sinuosa* |
| *carpatica* |  | 80.8-84.0 | 82.5-84.4 | 82.9-84.6 |
| *irregulare* |  |  | 81.1-84.6 | 72.2-84.6 |
| *neocaledonica* |  |  |  | 83.7-83.9 |

***RPB1*:** *Durabilispora carpatica* PX570053, PX570054; *Rhizoglomus irregulare*

HG315984, HG315987; *Sclerocystis sinuosa* HG315990; *Silvaspora neocaledonica*

MW541061, MW541060.

**45S:** *Durabilispora carpatica* PX641487‒PX641493; *Rhizoglomus irregulare*

FJ235567, FJ235569, FM992381, FM865617, FR750070, FR750193; *Silvaspora*

*neocaledonica* KY362436, KY362437; *Sclerocystis sinuosa*

AJ133706+AJ437106+FJ461846, PQ459440, PQ459441.

**Supplementary table S2.** Percent genetic identities between the sequences of five exons and four introns of the *rpb1* gene and the 45S nuc rDNA

region of nine *Dominikia* species.

| **Intron 1** | | | | | | | | | |
| --- | --- | --- | --- | --- | --- | --- | --- | --- | --- |
|  | *achra* | *aurea* | *duoreactiva* | *iranica* | *lithuanica* | *difficilevidera* | *disticha* | *paraminuta* | *tatrensis* |
| *achra* |  | 86.2 | 90.2 | 79.5 | 86.7 | 83.8 | 81.7 | 84.2 | 85.7 |
| *aurea* |  |  | 90.2 | 85.2 | 85.2 | 89.5 | 86.6 | 90.7 | **93.9** |
| *duoreactiva* |  |  |  | 81.9 | 87.5 | 86.2 | 82.9 | 87.5 | 89.4 |
| *iranica* |  |  |  |  | 77.6 | 83.6 | 93.4 | 84.8 | 85.8 |
| *lithuanica* |  |  |  |  |  | 83.2 | 79.1 | 81.6 | 85.0 |
| *difficilevidera* |  |  |  |  |  |  | 86.2 | 88.3 | 88.2-88.3 |
| *disticha* |  |  |  |  |  |  |  | 85.8 | 86.5-87.2 |
| *paraminuta* |  |  |  |  |  |  |  |  | 88.7 |
| **Exon 3** | | | | | | | | | |
|  | *achra* | *aurea* | *duoreactiva* | *iranica* | *lithuanica* | *difficilevidera* | *disticha* | *paraminuta* | *tatrensis* |
| *achra* |  | 95.6-96.3 | 98.3-99.0 | 96.6-97.3 | 98.3-99.0 | 97.0-97.6 | 96.6-97.6 | 96.6-97.3 | 96.3-97.0 |
| *aurea* |  |  | 96.6 | 96.3 | 96.0 | 97.3 | 96.3-97.3 | 97.6 | **95.3-98.6** |
| *duoreactiva* |  |  |  | 97.6 | 98.6 | 97.3 | 97.0-98.0 | 97.6 | 94.6-97.3 |
| *iranica* |  |  |  |  | 97.0 | 97.0 | 98.3-99.0 | 97.3 | 94.3-97.0 |
| *lithuanica* |  |  |  |  |  | 97.3 | 96.3-97.3 | 97.0 | 94.0-96.6 |
| *difficilevidera* |  |  |  |  |  |  | 97.6-98.0 | 98.3 | 94.6-97.3 |
| *disticha* |  |  |  |  |  |  |  | 97.3-98.3 | 94.3-98.0 |
| *paraminuta* |  |  |  |  |  |  |  |  | 95.0-97.6 |
| **Exon 5** | | | | | | | | | |
|  | *achra* | *aurea* | *duoreactiva* | *iranica* | *lithuanica* | *difficilevidera* | *disticha* | *paraminuta* | *tatrensis* |
| *achra* |  | 97.0 | 99.3 | 96.2 | 99.5-99.7 | 97.7-97.9 | 97.0 | 96.8-97.0 | 96.6-96.8 |
| *aurea* |  |  | 96.8 | 95.6 | 96.6-96.8 | 96.8-97.0 | 96.6 | 96.4-96.6 | **99.5-99.7** |
| *duoreactiva* |  |  |  | 96.0 | 98.9-**99.1** | 97.5-97.7 | 96.8 | 96.8-97.0 | 96.4-96.6 |
| *iranica* |  |  |  |  | 95.8-96.0 | 95.8-96.0 | 97.2 | 95.4-95.6 | 95.4 |
| *lithuanica* |  |  |  |  |  | 97.5-97.7 | 96.6-96.8 | 96.6-97.0 | 96.2-96.6 |
| *difficilevidera* |  |  |  |  |  |  | 96.4-96.6 | 96.8-97.0 | 96.6-96.8 |
| *disticha* |  |  |  |  |  |  |  | 96.0-96.2 | 96.2-96.4 |
| *paraminuta* |  |  |  |  |  |  |  |  | 96.0-96.4 |
| **Exon 4 plus Exon 5** | | | | | | | | | |
|  | *achra* | *aurea* | *duoreactiva* | *iranica* | *lithuanica* | *difficilevidera* | *disticha* | *paraminuta* | *tatrensis* |
| *achra* |  | 97.4 | 99.4 | 96.9 | 99.6-99.7 | 97.7-97.9 | 97.4 | 97.7-97.8 | 97.2-97.3 |
| *aurea* |  |  | 97.3 | 97.0-98.1 | 97.3-97.4 | 97.0-97.4 | 97.7 | 97.4-97.6 | **99.7-99.8** |
| *duoreactiva* |  |  |  | 96.8 | 99.3-99.4 | 97.6-97.8 | 97.3 | 97.7-97.8 | 97.1-97.2 |
| *iranica* |  |  |  |  | 96.8-96.9 | 96.6-96.9 | 98.1 | 96.8-96.9 | 96.8-96.9 |
| *lithuanica* |  |  |  |  |  | 97.7-97.9 | 97.3-97.4 | 97.7-97.9 | 97.1-97.3 |
| *difficilevidera* |  |  |  |  |  |  | 97.0-97.2 | 97.7-97.8 | 97.0-97.3 |
| *disticha* |  |  |  |  |  |  |  | 97.2-97.3 | 97.4-97.6 |
| *paraminuta* |  |  |  |  |  |  |  |  | 97.3-97.4 |
| **Exons 1‒5** | | | | | | | | | |
|  | *achra* | *aurea* | *duoreactiva* | *iranica* | *lithuanica* | *difficilevidera* | *disticha* | *paraminuta* | *tatrensis* |
| *achra* |  | 96.4-96.6 | 99.0-99.1 | 96.4-96.6 | 99.3-99.5 | 96.9-97.2 | 96.7-97.0 | 97.2-97.4 | 96.5-96.7 |
| *aurea* |  |  | 96.5-96.9 | 96.6-98.6 | 96.5-96.9 | 96.7-97.1 | 97.1-97.2 | 97.1-97.2 | **99.5-99.6** |
| *duoreactiva* |  |  |  | 98.9-99.1 | 98.9-99.1 | 96.8-97.1 | 96.8-96.9 | 97.4 | 96.5-96.7 |
| *iranica* |  |  |  |  | 96.4-96.5 | 96.5-96.7 | 98.5-98.7 | 96.7-96.9 | 96.7-96.8 |
| *lithuanica* |  |  |  |  |  | 97.0-97.2 | 96.7-96.9 | 97.3 | 96.6-96.7 |
| *difficilevidera* |  |  |  |  |  |  | 96.8-97.2 | 97.5-97.7 | 96.7-96.9 |
| *disticha* |  |  |  |  |  |  |  |  | 97.2-97.4 |
| *paraminuta* |  |  |  |  |  |  |  |  |  |
| **Exons 1‒5 plus Introns 1‒4** | | | | | | | | | |
|  | *achra* | *aurea* | *duoreactiva* | *iranica* | *lithuanica* | *difficilevidera* | *disticha* | *paraminuta* | *tatrensis* |
| *achra* |  | 90.5-90.6 | 96.7-96.8 | 90.4-90.8 | 97.0-97.2 | 92.4-92.7 | 91.5-91.6 | 93.9-94.1 | 89.9-90.5 |
| *aurea* |  |  | 91.0-91.1 | 92.4-92.5 | 90.7 | 91.1-91.3 | 92.8-92.9 | 91.8-92.0 | **97.2-97.6** |
| *duoreactiva* |  |  |  | 90.9-91.2 | 96.8-96.9 | 93.4-93.6 | 91.7-91.9 | 94.6-94.7 | 90.3-90.9 |
| *iranica* |  |  |  |  | 90.5-90.8 | 91.2-91.6 | 97.2-97.6 | 92.2-92.3 | 91.8-92.5 |
| *lithuanica* |  |  |  |  |  | 93.93.1 | 91.5-91.6 | 94.0-94.1 | 90.1-90.6 |
| *difficilevidera* |  |  |  |  |  |  | 92.3-92.5 | 94.1-94.3 | 90.3-90.8 |
| *disticha* |  |  |  |  |  |  |  | 92.7-92.9 | 92.2-92.9 |
| *paraminuta* |  |  |  |  |  |  |  |  | 91.1-91.6 |
| **45S** | | | | | | | | | |
|  | *achra* | *aurea* | *duoreactiva* | *iranica* | *lithuanica* | *difficilevidera* | *disticha* | *paraminuta* | *tatrensis* |
| *achra* |  | 89.4-90.5 | 94.3-94.4 | 84.4-85.4 | 96.5-96.9 | 89.3-89.4 | 90.0-90.3 | 91.8-92.1 | 90.1-90.8 |
| *aurea* |  |  | 89.8-90.5 | 84.7-86.3 | 90.5-91.1 | 89.3-89.8 | 90.4-90.8 | 90.7-91.7 | **95.8-97.6** |
| *duoreactiva* |  |  |  | 83.8-84.4 | 95.0 | 90.3 | 90.2 | 91.8.-92.0 | 89.8-90.5 |
| *iranica* |  |  |  |  | 84.3-85.2 | 83.0-83.6 | 87.4-88.2 | 86.1-87.7 | 84.7-86.3 |
| *lithuanica* |  |  |  |  |  | 89.9-90.0 | 90.2-90.6 | 92.1-92.3 | 90.5-91.1 |
| *difficilevidera* |  |  |  |  |  |  | 88.7-88.8 | 90.5-90.6 | 89.3-89.8 |
| *disticha* |  |  |  |  |  |  |  | 84.0-90.9 | 90.3-90.8 |
| *paraminuta* |  |  |  |  |  |  |  |  | 91.1-91.7 |

**RPB1:** *Dominikia achra* KX784804, KX784805, KX784807, KX784806; *D. aurea* KX784792, KX784793; *D. bonfanteae* MW249067, MW249068;

*D. difficilevidera* KX784795, KX784796, KX784797; *D._disticha* KX784784, KX784785, KX784786, KX784787; *D. duoreactiva* KX784800, KX784801;

*D. gansuensis* MZ960429, MZ960433, MZ960431, MZ960432, MZ960430; *D. glomerocarpica* MW541064, MW541065; *D. iranica* KX784788, KX784789, KX784790, KX784791; *D. lithuanica* KX784769, KX784770, KX784771; *D. paraminuta* KX784799, KX784798; *D.* *tatrensis* PX570049‒PX570052.

**45S:** *Dominikia achra* KJ564157, KJ564159; *D. aurea* KM056657, KM056658, KM056659, KM056660, KM056661, KM056662, KM056663, KM056664;

*D. difficilevidera* KR105644, KR105646; *D. disticha* KJ564146, KJ564147; *D. duoreactiva* KR105638, KR105641; *D. iranica* KJ564153, KJ564156;

*D. lithuanica* KX758115, KX758119; *D. paraminuta* KJ564166, KJ564169, KJ564168; *D. tatraensis* PX641494‒PX641499.

**Supplementary table S3.** Percent genetic identities between the sequences of intron 1, exon 3, exons 4

and 5, exons 1‒5, exons 1‒5 plus introns 1‒4 of the *rpb1* gene and the 45S nuc rDNA region of five *Glomus*

species.

| **Intron 1** | | | | | |
| --- | --- | --- | --- | --- | --- |
|  | *atlanticum* | *bareae* | *macrocarpum* | *rugosae* | *tetrastratosum* |
| *atlanticum* |  | 89.4 | 95.3 | 94.4-94.8 | 89.7 |
| *bareae* |  |  | 86.6 | 85.4-85.7 | 95.9 |
| *macrocarpum* |  |  |  | 91.4-91.8 | 86.9 |
| *rugosae* |  |  |  |  | 86.4-86.8 |
| **Exon 3** | | | | | |
|  | *atlanticum* | *bareae* | *macrocarpum* | *rugosae* | *tetrastratosum* |
| *atlanticum* |  | 97.5 | **99.6** | **100** | 97.5 |
| *bareae* |  |  | 97.8 | 97.5 | 98.7 |
| *macrocarpum* |  |  |  | **99.6** | 97.8 |
| *rugosae* |  |  |  |  | 97.5 |
| **Exon 4 plus Exon 5** | | | | | |
|  | *atlanticum* | *bareae* | *macrocarpum* | *rugosae* | *tetrastratosum* |
| *atlanticum* |  | 97.7-97.8 | 99.0-99.1 | 99.2-99.3 | 97.8-98.0 |
| *bareae* |  |  | 97.7-97.8 | 97.9-98.0 | 99.4-99.6 |
| *macrocarpum* |  |  |  | 99.4 | 97.9-98.0 |
| *rugosae* |  |  |  |  | 98.1-98.2 |
| **Exons 1‒5** | | | | | |
|  | *atlanticum* | *bareae* | *macrocarpum* | *rugosae* | *tetrastratosum* |
| *atlanticum* |  | 97.6-97.8 | 99.0-99.1 | 99.2-99.3 | 97.8-97.9 |
| *bareae* |  |  | 97.7-97.8 | 97.9-98.0 | 99.5 |
| *macrocarpum* |  |  |  | 99.5 | 97.8-97.9 |
| *rugosae* |  |  |  |  | 98.1 |
| **Exons 1‒5 plus Introns 1‒4** | | | | | |
|  | *atlanticum* | *bareae* | *macrocarpum* | *rugosae* | *tetrastratosum* |
| *atlanticum* |  | 94.5-94.6 | 98.1 | 98.2-98.4 | 94.2-94.3 |
| *bareae* |  |  | 94.0-94.1 | 94.2-94.3 | 97.1-97.2 |
| *macrocarpum* |  |  |  | 97.6-97.8 | 94.5-94.6 |
| *rugosae* |  |  |  |  | 93.7-93.9 |
| **45S** | | | | | |
|  | *atlanticum* | *bareae* | *macrocarpum* | *rugosae* | *tetrastratosum* |
| *atlanticum* |  | 89.1-89.8 | 94.1-95.1 | 95.1-96.5 | 86.5-89.4 |
| *bareae* |  |  | 89.1-89.8 | 88.8-90.1 | 92.4-96.6 |
| *macrocarpum* |  |  |  | 95.0-96.3 | 87.3-89.0 |
| *rugosae* |  |  |  |  | 87.5-89.0 |

***RPB1*:** *Glomus atlanticum* MW249069, MW249071; *G. rugosae rugosae* PP556328, PP556329,

*G. macrocarpum* HG316021; *G. bareae* MH560593, MH560595, MH560594; *G. tetrastratosum* MG710515,

MG710516.

**45S:** *Glomus atlanticum* MW232911, MW232912, MW232913; *G. bareae* MH560607, MH560608,

*G. macrocarpum* FR750526, FR750529, FR750530, FR750532; *G. rugosae* P532762, PP532763, PP532764,

PP532765, PP532766, PP532767; *G. tetrastratosum* KM056651, KM056655, KM056656.
