# Supplementary material for: A new genus (Durabilispora) and two new species (D. carpatica, Dominikia tatrensis) in Glomerales (Glomeromycota)
Source: MycoKeys. 2026 Jun 24;134:313–40. doi: 10.3897/mycokeys.134.187344 (PMC13324476; doi:10.3897/mycokeys.134.187344)
Supplement: Supplementary material 8 — Details of isolates [file mycokeys-134-313-s008.docx]

**Supplementary material 8.** Details of isolates included in the molecular study.

| Species | Strain | GenBank accession numbers | | | |  |
| --- | --- | --- | --- | --- | --- | --- |
|  |  | 45S | ITS1-5.8S-ITS2_ | 28S | *rpb1* | References |
| *Blaszkowskia deserticola* | BEG73-Hsp30-11+ BEG73-HSp30-15 | JQ048840+JQ048860 | N* | N | N | da Silva et al. 2023 |
| *Blaszkowskia deserticola* | BEG73-HSp30-6+BEG7-3-HSp30-6 | JQ048829+JQ048857 | N | N | N | da Silva et al. 2023 |
| *Blaszkowskia deserticola* | BEG73-HSp30-12+ BEG73-HSp30-19 | JQ048838+JQ048859 | N | N | N | da Silva et al. 2023 |
| *Complexispora multistratosa* | 455s1 | OQ437298 | N | N | OQ435059 | Błaszkowski et al. 2023 |
| *Complexispora multistratosa* | 455s9 | OQ437304 | N | N | OQ435058 | Błaszkowski et al. 2023 |
| *Delicatispora indica* | ITS06 | N | GU059544 | N | N | Błaszkowski et al. 2010 |
| *Delicatispora indica* | ITS24 | N | GU059547 | N | N | Błaszkowski et al. 2010 |
| *Delicatispora indica* | ITS08 | N | GU059548 | N | N | Błaszkowski et al. 2010 |
| *Delicatispora indica* | ITS14 | N | GU059549 | N | N | Błaszkowski et al. 2010 |
| *Dominikia achra* | 146-1-1 | KJ564157 | N | N | KX784804 | Błaszkowski et al. 2009, 2016 |
| *Dominikia achra* | 146-1-52 | KJ564159 | N | N | KX784806 | Błaszkowski et al. 2009, 2016 |
| *Dominikia aurea* | Da-1 | KM056657 | N | N | KX784792 | Błaszkowski et al. 2016 |
| *Dominikia aurea* | Da-11 | KM056658 | N | N | N | Błaszkowski et al. 2016 |
| *Dominikia aurea* | Da-2 | KM056659 | N | N | KX784793 | Błaszkowski et al. 2016 |
| *Dominikia aurea* | Da-21 | KM056660 | N | N | N | Błaszkowski et al. 2016 |
| *Dominikia aurea* | Da-22 | KM056661 | N | N | N | Błaszkowski et al. 2016 |
| *Dominikia aurea* | Da-3 | KM056662 | N | N | N | Błaszkowski et al. 2016 |
| *Dominikia aurea* | Da-31 | KM056663 | N | N | N | Błaszkowski et al. 2016 |
| *Dominikia aurea* | Da-32 | KM056664 | N | N | N | Błaszkowski et al. 2016 |
| *Dominikia bernensis* | FO310- 310.2 | HG938302 | N | N | N | Oehl et al. 2015 |
| *Dominikia bernensis* | FO310- 310.5 | HG938304 | N | N | N | Oehl et al. 2015 |
| *Dominikia bonfanteae* | Db_33 | MW232905 | N | N | MW249068 | Błaszkowski et al. 2021 |
| *Dominikia bonfanteae* | Db_3 | MW232906 | N | N | MW249067 | Błaszkowski et al. 2021 |
| *Dominikia difficilevidera* | 279-101 | KR105644 | N | N | KX784795 | Błaszkowski et al. 2015, 2016 |
| *Dominikia difficilevidera* | 279-103 | KR105646 | N | N | KX784797 | Błaszkowski et al. 2015, 2016 |
| *Dominikia disticha* | 237-1-88 | KJ564146 | N | N | KX784784 | Błaszkowski et al. 2015, 2016 |
| *Dominikia disticha* | 237-1-21 | KJ564147 | N | N | KX784787 | Błaszkowski et al. 2015, 2016 |
| *Dominikia duoreactiva* | 271-10 | KR105638 | N | N | KX784800 | Błaszkowski et al. 2015, 2016 |
| *Dominikia duoreactiva* | 271-11 | KR105641 | N | N | KX784801 | Błaszkowski et al. 2015, 2016 |
| *Dominikia gansuensis* | DG-11 | *MZ448287* | N | N | MZ960429 | Yu et al. 2022 |
| *Dominikia gansuensis* | DG-14 | MZ448290 | N | N | MZ960430 | Yu et al. 2022 |
| *Dominikia glomerocarpica* | Dg_2 | MW507148 | N | N | MW541064 | Błaszkowski et al. 2021 |
| *Dominikia glomerocarpica* | Dg_6 | MW507152 | N | N | MW541065 | Błaszkowski et al. 2021 |
| *Dominikia iranica* | 187-2-1-13 | KJ564153 | N | N | KX784788 | Błaszkowski et al. 2010, 2016 |
| *Dominikia iranica* | 187-2-2-23 | KJ564156 | N | N | KX784791 | Błaszkowski et al. 2010, 2016 |
| *Dominikia lithuanica* | 331-1 | KX758115 | N | N | KX784769 | Błaszkowski et al. 2010, 2016 |
| *Dominikia lithuanica* | 331-7 | KX758119 | N | N | KX784770 | Błaszkowski et al. 2010, 2016 |
| *Dominikia minuta* | 137-2-25 | KJ564163 | N | N | N | Błaszkowski et al. 2015 |
| *Dominikia minuta* | 137-2-15 | KJ564165 | N | N | N | Błaszkowski et al. 2015 |
| *Dominikia paraminuta* | 211-1-53 | KJ564166 | N | N | KX784799 | Błaszkowski et al. 2025 |
| *Dominikia paraminuta* | 211-1-32 | KJ564168 | N | N | KX784798 | Błaszkowski et al. 2025 |
| *Dominikia paraminuta* | 211-1-43 | KJ564169 | N | N | N | Błaszkowski et al. 2025 |
| *Dominikia tatrensis* | 1 1 | PX641494 | N | N | PX570052 | This paper. |
| *Dominikia tatrensis* | 1 5 | PX641497 | N | N | N | This paper. |
| *Dominikia tatrensis* | 7 7 | PX641499 | N | N | N | This paper. |
| *Dominikia tatrensis* | 7 6 | PX641498 | N | N | PX570050 | This paper. |
| *Dominikia tatrensis* | 1 2 | PX641495 | N | N | PX570051 | This paper. |
| *Dominikia tatrensis* | 1 3 | PX641496 | N | N | PX570051 | This paper. |
| *Durabilispora carpatica* | 6 1 | PX641492 | N | N | PX570054 | This paper. |
| *Durabilispora carpatica* | 3 4 | PX641489 | N | N | N | This paper. |
| *Durabilispora carpatica* | 6 2 | PX641493 | N | N | N | This paper. |
| *Durabilispora carpatica* | 3 2 | PX641488 | N | N | N | This paper. |
| *Durabilispora carpatica* | 5 | PX641490 | N | N | N | This paper. |
| *Durabilispora carpatica* | 1 | PX641487 | N | N | PX570053 | This paper. |
| *Durabilispora carpatica* | 6 | PX641491 | N | N | N | This paper. |
| *Entrophospora claroidea* | CA-OT-126-3-2- pHS112-40 | FN547625 | N | N | N | Stockinger et al. 2010 |
| *Entrophospora claroidea* | CA-OT-126-3-2-pHS112-15 | FN547626 | N | N | N | Stockinger et al. 2010 |
| *Entrophospora claroidea* | CA-OT-126-3-2-pHS112-17 | FN547628 | N | N | N | Stockinger et al. 2010 |
| *Entrophospora claroidea* | Att1063-4-pCK085-8 | FR750061 | N | N | N | Krüger et al. 2012 |
| *Entrophospora claroidea* | Att1063-3/SW210-pHS035-33 | FR750074 | N | N | N | Krüger et al. 2012 |
| *Entrophospora claroidea* | Att1063-3/SW210-pHS035-44 | FR750076 | N | N | N | Krüger et al. 2012 |
| *Epigeocarpum crypticum* | Ec_10 | MW507153 | N | N | N | Błaszkowski et al. 2021 |
| *Epigeocarpum crypticum* | Ec_6 | MW507156 | N | N | N | Błaszkowski et al. 2021 |
| *Funneliformis mosseae* | BEG12-pHS110-38 | FN547476 | N | N | N | Stockinger et al. 2010 |
| *Funneliformis mosseae* | BEG12-pHS110-37 | FN547486 | N | N | N | Stockinger et al. 2010 |
| *Funneliglomus sanmartinensis* | 1b | MK348927 | N | N | N | Corazon-Guivin et al. 2019b |
| *Funneliglomus sanmartinensis* | 2b | MK348929 | N | N | N | Corazon-Guivin et al. 2019b |
| *Glomus macrocarpum* | W5288-pHS093-32 | FR750526 | N | N | HG316006 | Krüger et al. 2012; Stockinger et al. 2014 |
| *Glomus macrocarpum* | W5288-pHS093-48 | FR750529 | N | N | HG316007 | Krüger et al. 2012; Stockinger et al. 2014 |
| *Halonatospora pansihalos* | Hp2 | MH560600 | N | N | N | Błaszkowski et al. 2018b |
| *Halonatospora pansihalos* | Hp9 | MH560603 | N | N | N | Błaszkowski et al. 2018b |
| *Kamienskia bistrata* | 205-1-8 | KJ564134 | N | N | N | Błaszkowski et al. 2015 |
| *Kamienskia bistrata* | 205-1-7 | KJ564136 | N | N | N | Błaszkowski et al. 2015 |
| *Macrodominikia compressa* | Mc_4 | PQ464093 | N | N | N | Błaszkowski et al. 2025 |
| *Macrodominikia compressa* | Mc_6 | PQ464094 | N | N | N | Błaszkowski et al. 2025 |
| *Macrodominikia compressa* | FO352-352.3 | HG798896 | N | N | N | Oehl et al. 2014 |
| *Macrodominikia compressa* | FO352-352.4 | HG798897 | N | N | N | Oehl et al. 2014 |
| *Macrodominikia compressa* | FO352-352.5 | HG798898 | N | N | N | Oehl et al. 2014 |
| *Macrodominikia compressa* | ASV_432 | MT765726 | N | N | N | Oehl et al. 2014 |
| *Microdominikia litorea* | Dl-6 | MG710518 | N | N | N | Błaszkowski et al. 2018c |
| *Microdominikia litorea* | Dl-10 | MG710517 | N | N | N | Błaszkowski et al. 2018c |
| *Microdominikia litorea* | Dl-8 | MG710519 | N | N | N | Błaszkowski et al. 2018c |
| *Microdominikia litorea* | Dl-B1 | MG710520 | N | N | N | Błaszkowski et al. 2018c |
| *Microkamienskia divaricata* | 240-6 | KX758123 | N | N | N | Błaszkowski et al. 2016 |
| *Microkamienskia divaricata* | 240-10 | KX758125 | N | N | N | Błaszkowski et al. 2016 |
| *Microkamienskia perpusilla* | 169-3-1-11 | KJ564139 | N | N | N | Błaszkowski et al. 2009 |
| *Microkamienskia perpusilla* | 169-3-5-51 | KJ564142 | N | N | N | Błaszkowski et al. 2009 |
| *Microkamienskia peruviana* | 1 | MK903005 | N | N | N | Corazon-Guivin et al. 2019d |
| *Microkamienskia peruviana* | 2 | MK903006 | N | N | N | Corazon-Guivin et al. 2019d |
| *Microviscospora peruviscosa* | 1 | OQ396746 | N | N | N | Corazon-Guivin et al. 2023 |
| *Microviscospora peruviscosa* | 6 | OQ396751 | N | N | N | Corazon-Guivin et al. 2023 |
| *Nanoglomus plukenetiae* | 5 | MK875634 | N | N | N | Corazon-Guivin et al. 2019a |
| *Nanoglomus plukenetiae* | 8 | MK875637 | N | N | N | Corazon-Guivin et al. 2019a |
| *Oehlia diaphana* | Od_15 | MG836663 | N | N | N | Błaszkowski et al. 2018a |
| *Oehlia diaphana* | Od_6 | MG836664 | N | N | N | Błaszkowski et al. 2018a |
| *Oehlia diaphana* | Od_4 | MG836665 | N | N | N | Błaszkowski et al. 2018a |
| *Orientoglomus emiratium* | 344-3 | KY555051 | N | N | N | Al-Yahya’ei et al. 2017 |
| *Orientoglomus emiratium* | 344-5 | KY555053 | N | N | N | Al-Yahya’ei et al. 2017 |
| *Rhizoglomus arabicum* | F84-1 | KF154764 | N | N | N | Symanczik et al. 2014 |
| *Rhizoglomus arabicum* | F80-B | KF154767 | N | N | N | Symanczik et al. 2014 |
| *Rhizoglomus cacao* | ZL-2022b-1 | OM109241 | N | N | N | Corazon-Guivin et al. 2022 |
| *Rhizoglomus cacao* | ZL-2022b-2 | OM109242 | N | N | N | Corazon-Guivin et al. 2022 |
| *Rhizoglomus clarum* | AU402B-2 | N | N | FJ461823 | HG316001 | Stockinger et al. 2014 |
| *Rhizoglomus clarum* | BR152B | N | N | FJ461825 | HG316024 | Stockinger et al. 2014 |
| *Rhizoglomus dalpeae* | rd6 | MN130953 | N | N | MW249072 | Błaszkowski et al. 2019, 2021 |
| *Rhizoglomus dalpeae* | rd8 | MN130954 | N | N | MW249073 | Błaszkowski et al. 2019, 2021 |
| *Rhizoglomus dunense* | 232-1 | N | N | KY555039 | KY555047 | Al-Yahya’ei et al. 2017 |
| *Rhizoglomus dunense* | 232-2 | N | N | KY555040 | KY555050 | Al-Yahya’ei et al. 2017 |
| *Rhizoglomus fasciculatum* | MUCL 46100- pHS033-11 | FR750071 | N | N | N | Krüger et al. 2012 |
| *Rhizoglomus fasciculatum* | MUCL 46100- pHS033-2 | FR750072 | N | N | N | Krüger et al. 2012 |
| *Rhizoglomus intraradices* | MUCL52327 | HE817875 | N | N | N | Walker et al. 2021 |
| *Rhizoglomus intraradices* | Att4-64- pHS99-11 | FM865597 | N | N | N | Stockinger et al. 2009 |
| *Rhizoglomus intraradices* | Att4-64- pHS99-32 | FM865601 | N | N | N | Stockinger et al. 2009 |
| *Rhizoglomus intraradices* | Att 4-38- pHS089-15 | FM865566 | N | N | HG316020 | Stockinger et al. 2009, 2014 |
| *Rhizoglomus invermaium* | Att1646-ECU102P11 | HG969374 | N | N | N | Walker et al. 2021 |
| *Rhizoglomus invermaium* | Att1646-ECU102P24 | HG969375 | N | N | N | Walker et al. 2021 |
| *Rhizophagus invermaium* | Att1646-VP011-01 | HG969378 | N | N | N | Walker et al. 2021 |
| *Rhizoglomus invermaium* | Att1646-VP011-15 | HG969380 | N | N | N | Walker et al. 2021 |
| *Rhizophagus invermaium* | Att1646-ECU102P15 | HG969381 | N | N | N | Walker et al. 2021 |
| *Rhizoglomus invermaium* | Att1646-ECU102P12 | HG969382 | N | N | N | Walker et al. 2021 |
| *Rhizoglomus invermaium* | Att1646-ECU102P28 | HG969383 | N | N | N | Walker et al. 2021 |
| *Rhizoglomus invermaium* | Att1646-ECU102P14 | HG969384 | N | N | N | Walker et al. 2021 |
| *Rhizoglomus invermaium* | Att1646-ECU102P19 | HG969385 | N | N | N | Walker et al. 2021 |
| *Rhizoglomus invermaium* | Att1646-ECU102P21 | HG969386 | N | N | N | Walker et al. 2021 |
| *Rhizoglomus invermaium* | Att1646-ECU102P22 | HG969391 | N | N | N | Walker et al. 2021 |
| *Rhizoglomus invermaium* | Att1646-ECU102P25 | HG969392 | N | N | N | Walker et al. 2021 |
| *Rhizoglomus invermaium* | Att1646-ECU102P9 | HG969377 | N | N | N | Walker et al. 2021 |
| *Rhizoglomus irregulare* | MUCL43194- rLU_11 | N | N | FJ235569 | N | Boon et al. 2010 |
| *Rhizoglomus irregulare* | MUCL43194- rLU_17 | N | N | FJ235567 | HG315984 | Boon et al. 2010; Stockinger et al. 2014 |
| *Rhizoglomus irregulare* | Att690-23 | FM992381 | N | N | HG315987 | Stockinger et al. 2009, 2014 |
| *Rhizoglomus irregulare* | Att1192-27 | FM865617 | N | N | N | Stockinger et al. 2009 |
| *Rhizoglomus irregulare* | DAOM197198 | FR750070 | N | N | N | Krüger et al. 2012 |
| *Rhizoglomus irregulare* | "from Poland (trap culture)" | FR750193 | N | N | HG316002 | Krüger et al. 2012; Stockinger et al. 2014 |
| *Rhizoglomus maiae* | rm1 | MN130955 | N | N | N | Błaszkowski et al. 2019 |
| *Rhizoglomus maiae* | rm6 | MN130957 | N | N | N | Błaszkowski et al. 2019 |
| *Rhizoglomus melanum* | Dovre 6K-2 | HG964396 | N | N | N | Sudová et al. 2015 |
| *Rhizoglomus melanum* | Dovre 6K-4 | HG964397 | N | N | N | Sudová et al. 2015 |
| *Rhizoglomus melanum* | Dovre 6K-5 | HG964398 | N | N | N | Sudová et al. 2015 |
| *Rhizoglomus melanum* | Dovre 6K-6 | HG964399 | N | N | N | Sudová et al. 2015 |
| *Rhizophagus melanum* | Dovre 6K-7 | HG964400 | N | N | N | Sudová et al. 2015 |
| *Rhizoglomus natalensis* | 261-4 | N | N | KJ210826 | N | Błaszkowski et al. 2014a |
| *Rhizoglomus natalensis* | 261-6 | N | N | KJ210828 | N | Błaszkowski et al. 2014a |
| *Rhizoglomus proliferum* | MUCL41827- pHS113-33 | FM992398 | N | N | AM284981 | Stockinger et al. 2009; Redecker and Raab 2006 |
| *Rhizoglomus proliferum* | MUCL41827- pHS117-IID14 | FM992402 | N | N | AM284982 | Stockinger et al. 2009; Redecker and Raab 2006 |
| *Rhizoglomus silesianum* | rs4 | MN130960 | N | N | MW541063 |  |
| *Rhizoglomus silesianum* | rs8 | MN130961 | N | N | MW541062 | Błaszkowski et al. 2019, 2021 |
| *Rhizoglomus* *variabile* | Rv3 | MN384872 | N | N | N | Corazon-Guivin et al. 2019c |
| *Rhizoglomus variabile* | Rv4 | MN384873 | N | N | N | Corazon-Guivin et al. 2019c |
| *Rhizoglomus venetianum* | AT-2018- Rvd6 | LS974597 | N | N | N | Turrini et al. 2018 |
| *Rhizoglomus venetianum* | AT-2018- Rvd7 | LS974598 | N | N | N | Turrini et al. 2018 |
| *Rhizoglomus vesiculiferum* | Rv_16 | MG836660 | N | N | MG836650 | Błaszkowski et al. 2018 |
| *Rhizoglomus vesiculiferum* | Rv_10 | MG836659 | N | N | MG83665 | Błaszkowski et al. 2018 |
| *Sclerocarpum amazonicum* | Sa2 | MK036781 | N | N | MK036773 | Jobim et al. 2019 |
| *Sclerocarpum amazonicum* | Sa6 | MK036783 | N | N | N | Jobim et al. 2019 |
| *Sclerocystis sinuosa* | MD126  +MD126-21-2+ MD126.II.1.Q24RG2 | AJ133706+AJ437106+FJ461846 | N | N | N | Redecker et al. 2000; Bidartondo et al. 2002; Błaszkowski et al. 2021 |
| *Sclerocystis sinuosa* | Ss_1 | PQ459440 | N | N | HG315990 | Błaszkowski et al. 2025; Stockinger et al. 2014 |
| *Sclerocystis sinuosa* | Ss_2 | PQ459441 | N | N | N | Błaszkowski et al. 2025 |
| *Septoglomus turnauae* | 243-2 | KF060324 | N | N | N | Błaszkowski et al. 2014b |
| *Septoglomus turnauae* | 243-3 | KF060326 | N | N | N | Błaszkowski et al. 2014b |
| *Silvaspora neocaledonica* | rh1 | KY362436 | N | N | MW541060 | Błaszkowski et al. 2021 |
| *Silvaspora neocaledonica* | rh2 | KY362437 | N | N | MW541061 | Błaszkowski et al. 2021 |
